# One‐Step Syntheses of Face‐Centered Cubic Os_x_Pt_1‐x_/C with Near‐Zero‐Overpotential Hydrogen Evolution from Electronic‐State Engineering

**DOI:** 10.1002/advs.202504161

**Published:** 2025-05-11

**Authors:** Junyun Gao, Bo Huang

**Affiliations:** ^1^ School of Chemical Engineering and Technology Western China Science and Technology Innovation Harbor Xi'an Jiaotong University Xixian‐ward Xi'an 712000 China; ^2^ Shaanxi Tianyi Element Technology Co., Ltd High‐tech Industrial Development Zone Xianyang 712000 China; ^3^ School of Future Technology Western China Science and Technology Innovation Harbor Xi'an Jiaotong University Xixian‐ward Xi'an 712000 China; ^4^ National Innovation Platform (Center) for Industry‐Education Integration of Energy Storage Technology Western China Science and Technology Innovation Harbor Xi'an Jiaotong University Xixian‐ward Xi'an 712000 China

**Keywords:** electronic states engineering, hydrogen evolution reaction, near‐zero overpotential, one‐step in situ polyol method, OsPt solid solutions

## Abstract

The loading states and crystal structure control of solid solution catalysts, which greatly influence the catalytic performance, have not yet been achieved simultaneously due to the limitations of previous synthetic methodologies. In this work, a one‐pot in situ polyol method is developed for the phase‐control synthesis of face‐centered cubic (fcc)‐dominated and well‐dispersed immiscible Os_x_Pt_1‐x_/C. Most fcc‐Os_x_Pt_1‐x_/C catalysts exhibit superior hydrogen evolution reaction (HER) catalytic activities compared to those of Pt/C catalysts. Remarkably, the overpotential of fcc‐Os_0.3_Pt_0.7_/C in 0.5 m H_2_SO_4_ at 10 mA·cm^−2^ is only 1.0 mV as the top record. In Os_0.5_Pt_0.5_/C and Os_0.3_Pt_0.7_/C, the weakening of H adsorption on Pt sites, resulting from electronic state adjustments induced by Os alloying, modify the reaction pathways by promoting H_2_ desorption from more favorable coupled sites, thereby achieving state‐of‐the‐art HER catalytic activities.

## Graphical abstract

A one‐step in situ polyol method is proposed to realize steady‐state syntheses and phase control of immiscible Os_x_Pt_1−x_/C catalysts with homogeneous atomic mixing states. The overpotential at 10 mA·cm^−2^ of face‐centered cubic (fcc)‐Os_0.3_Pt_0.7_/C in 0.5 m H_2_SO_4_ is only 1.0 mV as the top‐level HER catalyst. The superior HER catalytic activities of fcc‐Os_x_Pt_1−x_/C are attributed to the more efficient H_2_ desorption processes undergoing the modified HER pathways.

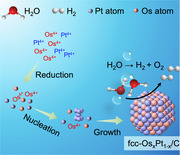

## 1 Introduction

Solid‐solution nanoalloys, in which the constituent elements are distributed homogeneously at the atomic level, have recently attracted significant attention, particularly in the field of catalysis due to their continuously adjustable multifunctional atomic sites and inherent electronic states across wide composition ranges.^[^ ^1^ ^]^ However, the solid solutions consisting of elements that cannot mix with each other in bulk states, referred to as immiscible solid solutions, could not be synthesized using traditional high‐temperature quenching methods,^[^ ^2^ ^]^ such as the Cu–Fe solid solution.^[^ ^3^ ^]^ Recently, chemical reduction methods have been widely employed for the syntheses of immiscible solid solutions, such as Cu–Ru,^[^ ^4^ ^]^ Ag–Rh,^[^ ^5^ ^]^ Au‐Ir^[^ ^6^ ^]^ and Ir‐Ru^[^ ^7^ ^]^ systems. For catalytic applications, depositing the as‐synthesized solid‐solution nanoparticles (NPs) on support materials typically requires additional post‐loading procedures, such as rotary evaporation,^[^ ^8^ ^]^ ultrasonication^[^ ^9^ ^]^ and grinding,^[^ ^10^ ^]^ which could not obtain homogeneously dispersed NPs on supports. Consequently, the degenerated catalytic performances caused by uneven loading states, resulting from the poorly controlled loading processes accordingly, have spurred the urgent need to develop the one‐step in situ synthetic methods for immiscible solid‐solution nanoalloys.

**Scheme 1:**
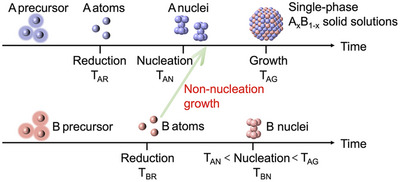
Reaction time analysis in the synthesis of single‐phase A_x_B_1‐x_ solid solutions.

For solid‐solution nanoalloys, the crystal structures mainly including face‐centered cubic (fcc), hexagonal close‐packed (hcp), and body‐centered cubic (bcc), greatly affect their physical and chemical properties, such as magnetism,^[^ ^11^ ^]^ sensing,^[^ ^12^ ^]^ catalysis,^[^ ^13^ ^]^ and so on.^[^ ^14^ ^]^ However, achieving simultaneous phase control and elimination of reduction rate differences for phase‐controlled synthesis of solid solutions still remains a significant challenge.^[^ ^15^ ^]^ This is because the nucleation time of the slower‐reduced element B must be precisely controlled to occur between the nucleation time and growth time of the faster‐reduced element A, enabling a non‐nucleation growth process to form single‐phase A_x_B_1‐x_ solid solutions (**Scheme** **1**). For these reasons, one‐step in situ synthetic methods that incorporate the phase control of solid solutions coexisting with supports have never been reported yet.

Hydrogen evolution reaction (HER) plays a key role in electrocatalytic H_2_ production techniques utilizing renewable energy sources such as wind‐powered and photovoltaic electricity, where Pt‐based materials are widely recognized as the best catalysts for HER.^[^ ^16^ ^]^ Very recently, Os‐based catalysts have demonstrated attractive HER activities,^[^ ^17^ ^]^ making Os a potential substitute for Pt‐based catalysts due to the high cost of Pt.^[^ ^18^ ^]^ Solid‐solution alloying of Os and Pt represents a rational approach to further boom the HER activities of Os‐ and Pt‐based catalysts, according to their extensive electronic states engineering. Furthermore, fcc‐structured Os_x_Pt_1‐x_ solid solutions exposing a large proportion of close‐packed (111) facets would provide very close H–H association sites, further shortening the reaction coordinates for H_2_ formation during HER.^[^ ^19^ ^]^ However, the promising HER catalysts as fcc‐dominated Os_x_Pt_1‐x_ solid solutions have not yet been synthesized.

Here we propose a one‐step in situ polyol (OIP) method, achieving in situ phase‐controlled synthesis of fcc‐dominated Os_x_Pt_1‐x_/C solid‐solution catalysts with uniform particle loadings and homogeneous elemental mixing states. Most fcc‐Os_x_Pt_1‐x_/C catalysts exhibited exceptionally high HER catalytic activities. Amazingly, the fcc‐Os_0.3_Pt_0.7_/C and fcc‐Os_0.5_Pt_0.5_/C demonstrated top‐level HER activities with extremely low overpotentials of 1.0 and 2.0 mV at 10 mA·cm^−2^ in 0.5 m H_2_SO_4_, respectively. Based on density functional theory (DFT) calculations, the excellent HER catalytic activities of fcc‐Os_x_Pt_1‐x_/C were attributed to the weaker H adsorptions, which undergo more energy‐favored reaction pathways due to electronic state tuning.

## 2 Results and Discussion

### 2.1 The Advantages of the One‐Step In Situ Polyol Method

**Figure 1:**
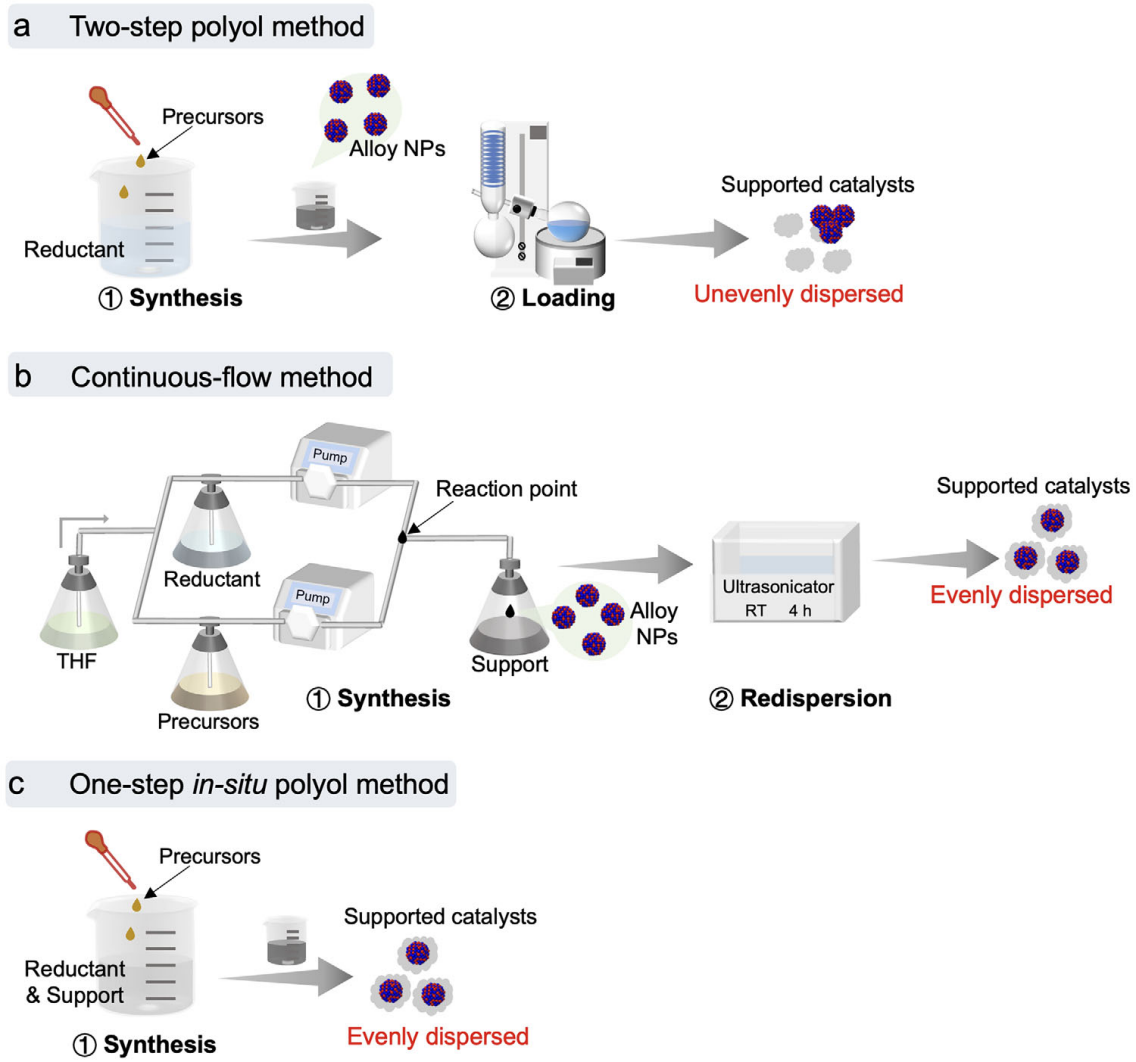
Synthetic methods comparison. a) Two‐step polyol method. b) Continuous‐flow method. c) One‐step in situ polyol method.

Immiscible solid solution catalysts were commonly synthesized using a two‐step polyol method,^[^ ^20^ ^]^ in which the NPs were first synthesized and then loaded onto support materials (**Figure** **1**a). However, this two‐step polyol method often results in uneven supporting states and requires the use of protecting agents, leading to reduced catalytic activities. Recently, the continuous‐flow method was proposed where the alloy NPs formed at the reaction point, and were subsequently dropped into solvent containing support materials (Figure 1b).^[^ ^9^, ^21^ ^]^ The solution was then sonicated to achieve better dispersion states. This method demands a complicated synthetic system and precise rate control to obtain homogeneous solid solutions. In this work, the Os_x_Pt_1‐x_/C (10 wt.%) catalysts were synthesized by the OIP method (Figure 1c), enabling the in situ synthesis of immiscible solid solutions on supports. Compared with the aforementioned methods, the OIP method ensures uniform particle distribution and integrates the synthesis process into a single step. Moreover, protecting agents such as PVP were not used in the OIP method, greatly simplifying the post‐synthetic processes and eliminating the detrimental effects of PVP on catalytic activities.

Notably, due to the similar reduction rates of K_2_OsCl_6_ and H_2_PtCl_6_·6H_2_O, fcc‐Os_x_Pt_1‐x_ NPs and hcp‐Os_x_Pt_1‐x_ NPs would be formed simultaneously by the conventional synthetic method, such as NaBH_4_ reduction method. To eliminate the influence of crystal structures on catalytic performance, we synthesized fcc‐Os_x_Pt_1‐x_/C catalysts using an optimized OIP method.

### 2.2 Syntheses and Characterizations of fcc‐Os_x_Pt_1‐x_/C

The synthetic process was described using Os_0.3_Pt_0.7_/C (with 10 wt.% loading amount) as an example: 9.2 mg K_2_OsCl_6_ and 93.2 mg H_2_PtCl_6_·6H_2_O were dissolved in a mixture of 5 mL diethylene glycol (DEG) and 2 mL H_2_O. Separately, 110 mL DEG solvent containing 77.1 mg acetylene black carbon support was degassed three times using liquid N_2_. The precursor solution was added dropwise within 2 min at 230 °C under a continuous N_2_ flow. The obtained solution was kept at 230 °C for another 5 min, followed by washing three times with ethyl acetate. The black powder was obtained after centrifugation. Os_x_Pt_1‐x_/C catalysts with other ratios (x = 0.1, 0.5, 0.7, 1) were synthesized by the same method (Table S1, Supporting Information). For comparison, Pt/C was prepared using different methods to obtain a similar NP size. The Os/Pt ratios of Os_x_Pt_1‐x_/C were determined by X‐ray fluorescence (XRF) measurements (Table S2, Supporting Information).

**Figure 2:**
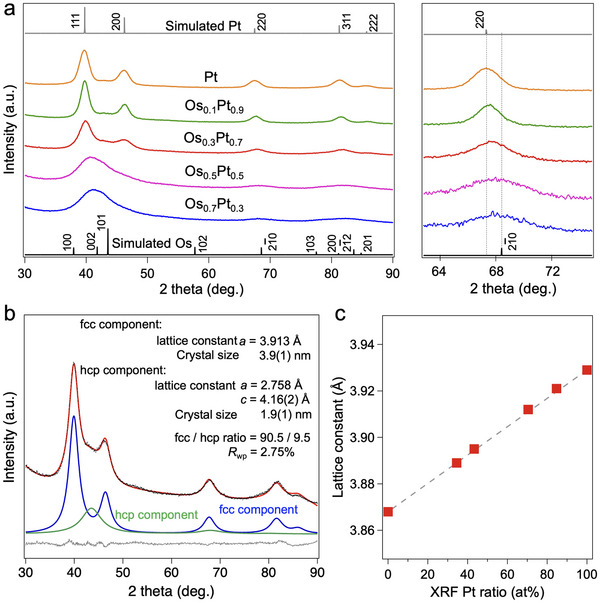
XRPD patterns and Rietveld refinement. a) XRPD patterns of the Os_x_Pt_1‐x_/C at 298 K. The radiation wavelength was 1.54056 Å. b) Rietveld refinement on XRPD pattern of Os_0.3_Pt_0.7_/C (black dots) and calculated pattern (red) at 303 K. The bottom line (gray) shows the difference profile. c) The lattice constants dependence versus XRF Pt ratio for Os_x_Pt_1−x_/C. The lattice constant value of fcc‐Os was simulated as 3.868 Å.

The crystal structures of Os_x_Pt_1‐x_/C were investigated by X‐ray powder diffraction (XRPD) measurements (**Figure** **2**). The Os_x_Pt_1‐x_/C primarily exhibited fcc‐crystal structures, with the {220} peaks shifting to higher angles as the Os content increased, indicating the alloy formations (Figure 2a). Rietveld refinements were performed on the XRPD patterns of Os_x_Pt_1‐x_/C (Figure 2b; Figures S1–S5, Supporting Information). For Os_0.3_Pt_0.7_/C, the fcc lattice constant *a* was determined as 3.913 Å, smaller than that of Pt (*a* = 3.923 Å) and larger than that of Os (*a* = 3.868 Å).^[^ ^22^ ^]^ The estimated fcc‐lattice constants of Os_x_Pt_1‐x_/C from Rietveld refinements showed a linear dependence on the Pt ratios obtained from XRF measurements, following Vegard's law, which may demonstrate the formations of Os_x_Pt_1‐x_ solid solutions (Figure 2c).

**Figure 3:**
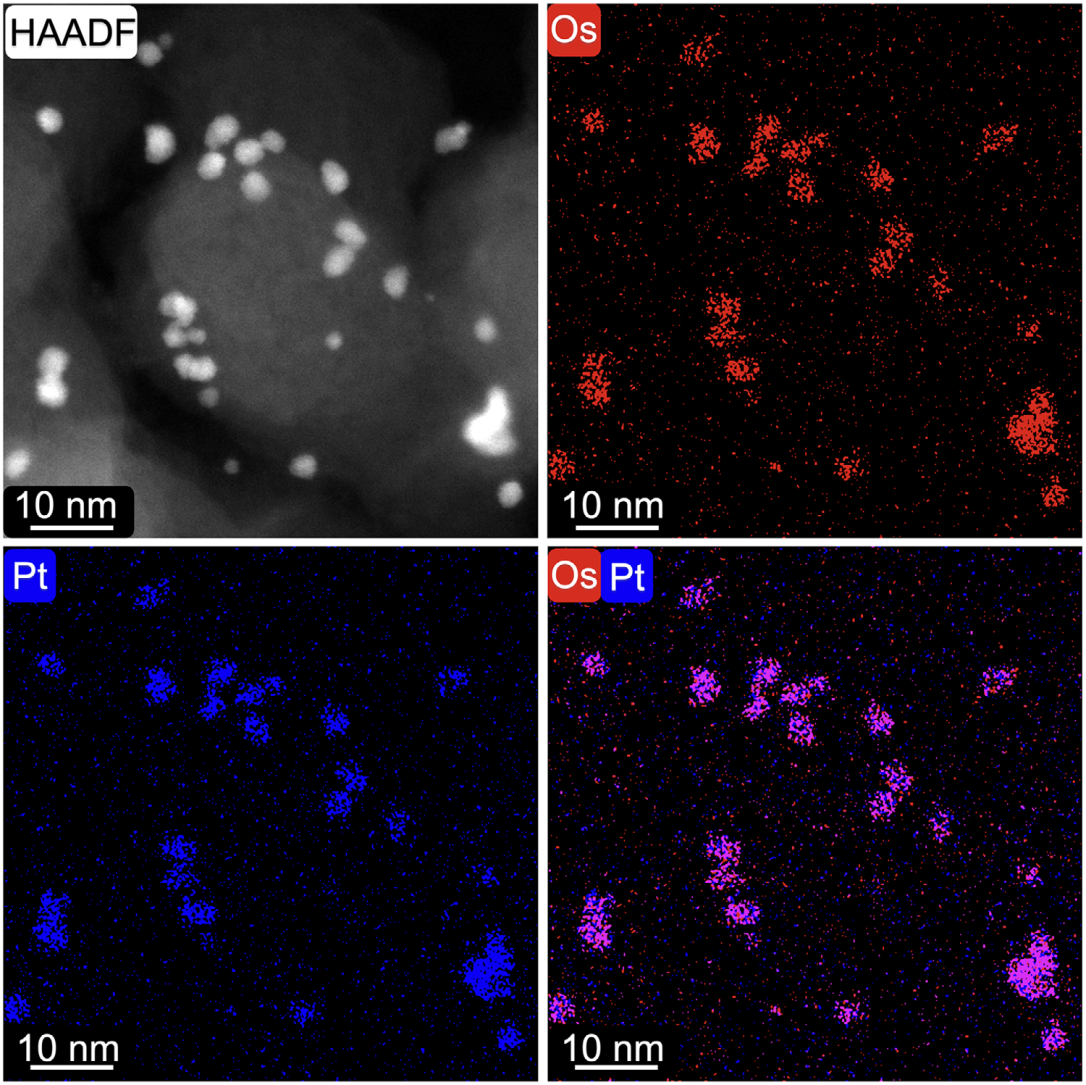
HAADF‐STEM image, Os L STEM‐EDX map (red), Pt L STEM‐EDX map (blue), and an overlay map of the Os and Pt distributions of Os_0.3_Pt_0.7_/C.

The supporting states of Os_x_Pt_1‐x_ NPs on carbon supports and mixing states of Os and Pt elements in each particle were evaluated by high‐angle annular dark‐field scanning transmission electron microscope (HAADF‐STEM) and STEM‐energy dispersive X‐ray (STEM‐EDX) mapping measurements. The HAADF image of Os_0.3_Pt_0.7_/C revealed the single dispersion of particles on acetylene black carbon support, and similar results were observed for Os_x_Pt_1‐x_/C with other ratios (**Figure** **3**; Figures S6–S12, Supporting Information). According to the HAADF images, the mean diameters of Os_x_Pt_1‐x_ NPs (x = 0, 0.1, 0.3, 0.5, 0.7, 1) were determined to be 5.1 ± 0.6, 5.3 ± 0.8, 3.2 ± 0.5, 2.6 ± 0.3, 3.7 ± 0.5, 1.5 ± 0.2 nm, respectively (Figures S6–S12, Supporting Information). The STEM‐EDX overlap map of Os_0.3_Pt_0.7_/C provided direct evidence for the homogeneous elemental distribution of Os and Pt within each particle (Figure 3). Similar results for Os_x_Pt_1‐x_/C with other ratios proved the formation of homogeneous Os_x_Pt_1‐x_ solid solutions on carbon supports (Figures S13–S16, Supporting Information). The Os/Pt ratios of Os_x_Pt_1‐x_/C were also calculated from EDX maps (Table S3, Supporting Information). The Os/Pt ratios of Os_x_Pt_1‐x_/C evaluated by EDX mapping, XRF measurements, and XRPD Rietveld refinements were all consistent with their nominal ratios, proving the accurate composition control (Table S4, Supporting Information).

### 2.3 Support and Reducibility Influences on One‐Step In Situ Polyol Method

**Figure 4:**
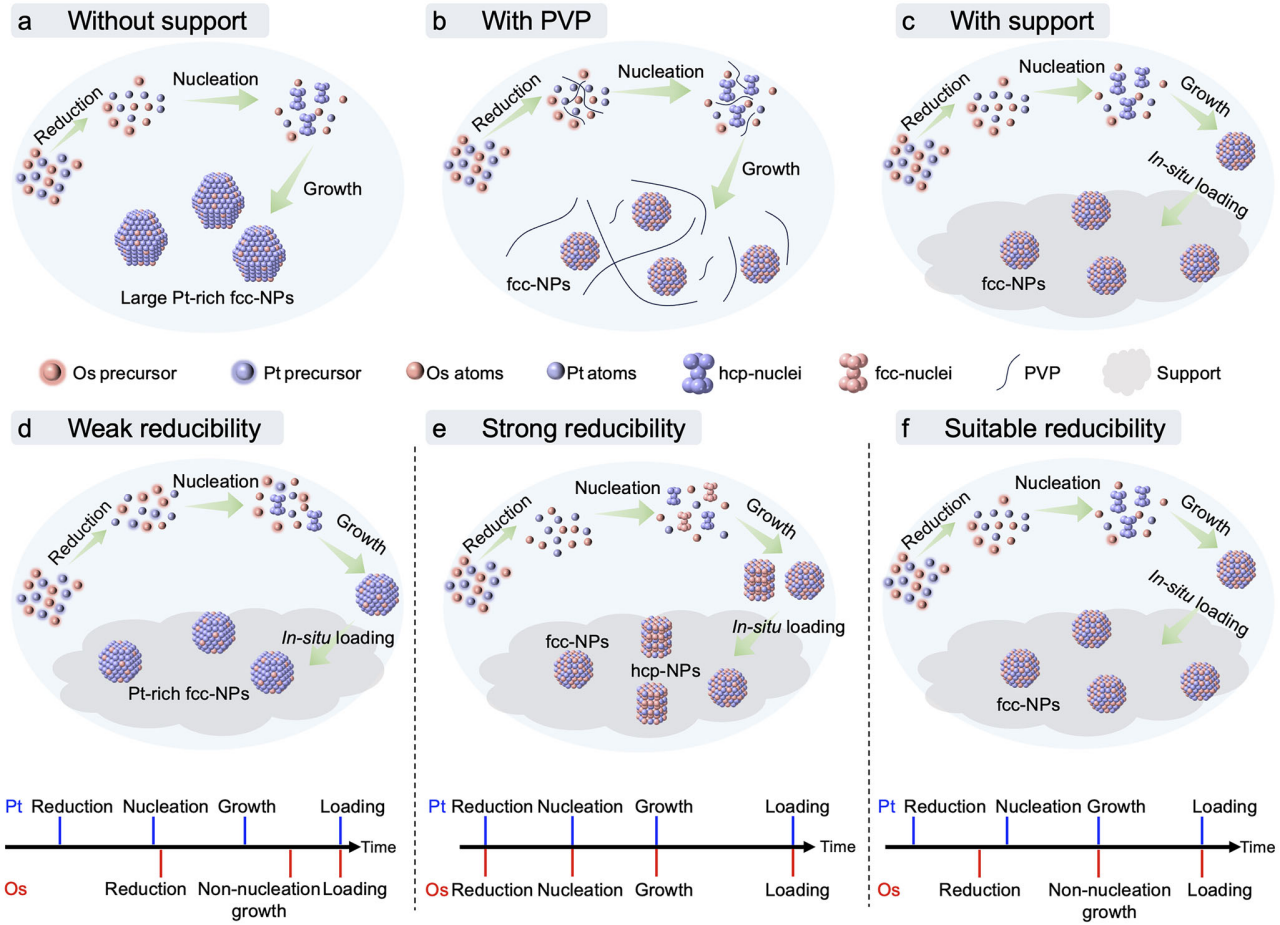
Synthetic mechanism analysis. The possible synthetic mechanisms in conditions a) without support, b) with PVP, and c) with support. The possible synthetic mechanisms and reaction time analysis in conditions with d) weak, e) strong, and f) suitable reducibility.

To specifically explain the successful syntheses of fcc‐Os_x_Pt_1‐x_/C, the influences of support materials and reducibility were investigated under different reaction conditions (synthesis details were shown in supporting information). Os_x_Pt_1‐x_ NPs without carbon support were also synthesized under the similar reaction condition (DEG at 230 °C) but with an obvious phase separation phenomenon as Pt‐rich large NPs (Figure S17, Supporting Information). Without the loading effect of support, the NPs concentration in the solution increased continuously, which boosted the more easily reduced Pt atoms to overgrow as Pt‐rich large NPs (**Figure** **4**a). This problem could be solved by the presence of PVP (Figure 4b). Therefore, similar to the role of PVP, the support materials can also prevent the undesirable overgrowth as steady‐state synthesis (continuous‐flow method also belongs to steady‐state synthesis),^[^ ^9^ ^]^ which enables the formation of single‐dispersed and homogeneously mixed Os_x_Pt_1‐x_ solid solutions in situ loaded on supports (Figure 4c).

We further investigated the influence of reducibility on one‐step in situ methods. When DEG was used as reductant at 180 °C with weak reducibility, Pt‐rich fcc‐Os_x_Pt_1‐x_/C catalyst was synthesized due to the insufficient reducibility of DEG at 180 °C for complete reduction of Os^4+^ (Figure 4d; Figure S18, Supporting Information). However, when reductants with strong reducibility such as NaBH_4_ were used, both hcp‐Os_x_Pt_1‐x_ NPs and fcc‐Os_x_Pt_1‐x_ NPs would be obtained, because excessive amounts of reduced Os and Pt atoms generated and nucleated independently (Figure 4e; Figure S19, Supporting Information).^[^ ^23^ ^]^ For fcc‐phase control, suitable reducibility may be necessary to ensure the nucleation time of Os stays between the nucleation time and growth time of Pt (Scheme 1).^[^ ^13^ ^]^ With suitable reducibility (DEG at 230 °C), H_2_PtCl_6_·6H_2_O was reduced to Pt atoms and formed fcc‐Pt nuclei rapidly, then the reduced Os atoms grew on the fcc‐Pt nuclei together with Pt atoms to form fcc‐Os_x_Pt_1‐x_ solid solutions loading on support (Figure 4f). The fcc‐Os_x_Pt_1‐x_ solid solutions formations confirmed nucleation times of Os staying between the nucleation times and growth times of Pt, and if nucleation times of Os were slower than the growth times of Pt, Pt‐Os core‐shell structure might be formed. Therefore, for the first time, we achieved the phase‐controlled steady‐state syntheses of the atomic‐level mixed solid solutions in situ loaded on supports for immiscible systems within one step in one pot, by precisely integrating support‐assisted overgrowth prevention and suitable reducibility in the OIP method.

In addition, the fcc‐Os_0.3_Pt_0.7_ with XC‐72R carbon as support was also synthesized successfully (Figures S20 and S21, Supporting Information), which demonstrated the universality of the OIP method. Other types of supports such as Al_2_O_3_, would also be suitable for the OIP method.

### 2.4 HER Catalytic Activities of fcc‐Os_x_Pt_1‐x_/C

**Figure 5:**
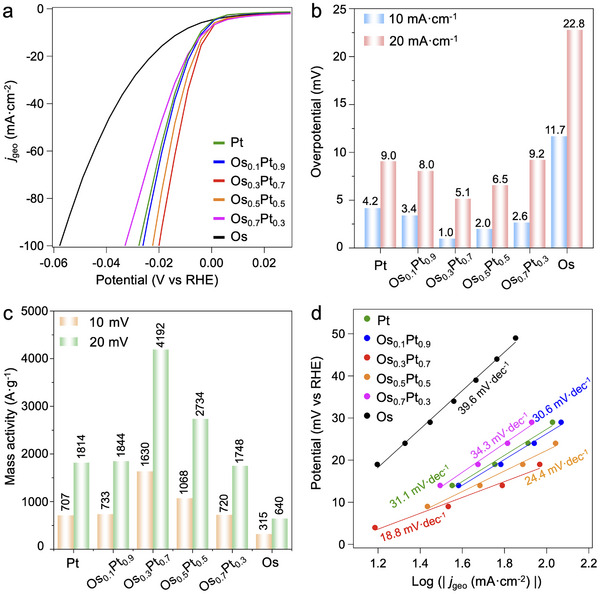
HER activities of Os_x_Pt_1‐x_/C in 0.5 m H_2_SO_4_. a) Polarization curves. b) Overpotentials at 10 mA·cm^−2^ (*η*_10_) and 20 mA·cm^−2^ (*η*_20_). c) Mass activities at overpotentials of 10 and 20 mV. d) Tafel plots.

The HER activities of fcc‐Os_x_Pt_1‐x_/C were investigated using a three‐electrode system. The Os_x_Pt_1‐x_/C catalysts were loaded onto glassy carbon electrodes with loading amounts of ≈35.4 µg_metal_·cm^−2^, as determined by XRF measurements (Table S5, Supporting Information), lower than most Pt‐based catalysts (Table S6, Supporting Information). The acidic HER catalytic activities of Os_x_Pt_1‐x_/C were evaluated in 0.5 m H_2_SO_4_ using linear scanning voltammetry (LSV) curves (**Figure** **5**a) and the overpotentials at 10 mA·cm^−2^ (*η*_10_) and 20 mA·cm^−2^ (*η*_20_), exhibited inverted volcano tendencies over Pt ratios (Figure 5b). Surprisingly, the *η*_10_ of Os_0.3_Pt_0.7_/C was only 1.0 mV, significantly outperforming recently reported Pt‐based catalysts (Table S6, Supporting Information). Furthermore, to quantitatively evaluate the HER activities, the mass activities of Os_x_Pt_1‐x_/C were calculated as current densities at 10/20 mV overpotentials normalized by metal masses. As shown in Figure 5c, Os_0.3_Pt_0.7_/C showed the highest mass activities at 10 and 20 mV overpotentials as 1630 and 4192 A·g_metal_^−1^, respectively, more than twice those of Pt/C (707 and 1814 A·g_metal_^−1^) and higher than most Pt‐based catalysts (Table S6, Supporting Information). Generally, small particle size contributes more to mass activities. In order to eliminate the influence of particle size on activities, the mass activities were further normalized by the proportion of surface atoms (Table S7, Supporting Information). Os_0.3_Pt_0.7_/C still showed the highest surface mass activities at 10 and 20 mV overpotentials as 6961 and 17900 A·g_surface_^−1^ (Figure S22, Supporting Information), surpassing those of Pt single‐atom catalysts (Table S8, Supporting Information), which demonstrated the state‐of‐the‐art intrinsic HER activity. Os_0.3_Pt_0.7_/C also showed greatly lower Tafel slop compared to Pt/C (Figure 5d), confirming its much faster kinetics.^[^ ^24^ ^]^ In addition, we compared the HER activities of Os_0.3_Pt_0.7_/C and commercial Pt/C, further validating the excellent HER activity of Os_0.3_Pt_0.7_/C (Figure S23, Supporting Information). The cost‐performances for Os_x_Pt_1‐x_/C catalysts were evaluated by mass activities based on the market prices of Os and Pt, and fcc‐Os_0.3_Pt_0.7_ still showed high cost‐performance as 155.9 A·g^−1^·dollar^−1^ at 20 mV overpotential, almost three times to that of pure Pt (Figure S24, Supporting Information).

The electrochemically active surface areas (ECSA) were determined by measuring cyclic voltammetry (CV) curves at scan rates of 20, 40, 60, 80, 100, and 120 mV·S^−1^, within the non‐faradaic regions from 0.2 to 0.27 mV. All Os_x_Pt_1‐x_/C (x = 0.1, 0.3, 0.5, 0.7) catalysts showed higher capacitances than Pt/C, consistent with their higher activities (Figures S25–S27, Supporting Information). The electrochemical impedance spectroscopy (EIS) tests were also conducted to further investigate the reaction kinetics (Figure S28, Supporting Information). The charge‐transfer resistance (*R*_ct_) of Os_0.3_Pt_0.7_/C (24.0 Ω) was lower than that of Pt/C (29.3 Ω), indicating faster charge transfer kinetics of Os_0.3_Pt_0.7_/C at the electrode interface during acidic HER process.^[^ ^25^ ^]^ To evaluate the HER stability, the chronoamperometry study was conducted for Os_0.5_Pt_0.5_/C, which still maintained higher activity than that of Pt/C after 20 h (Figure S29, Supporting Information).

**Figure 6:**
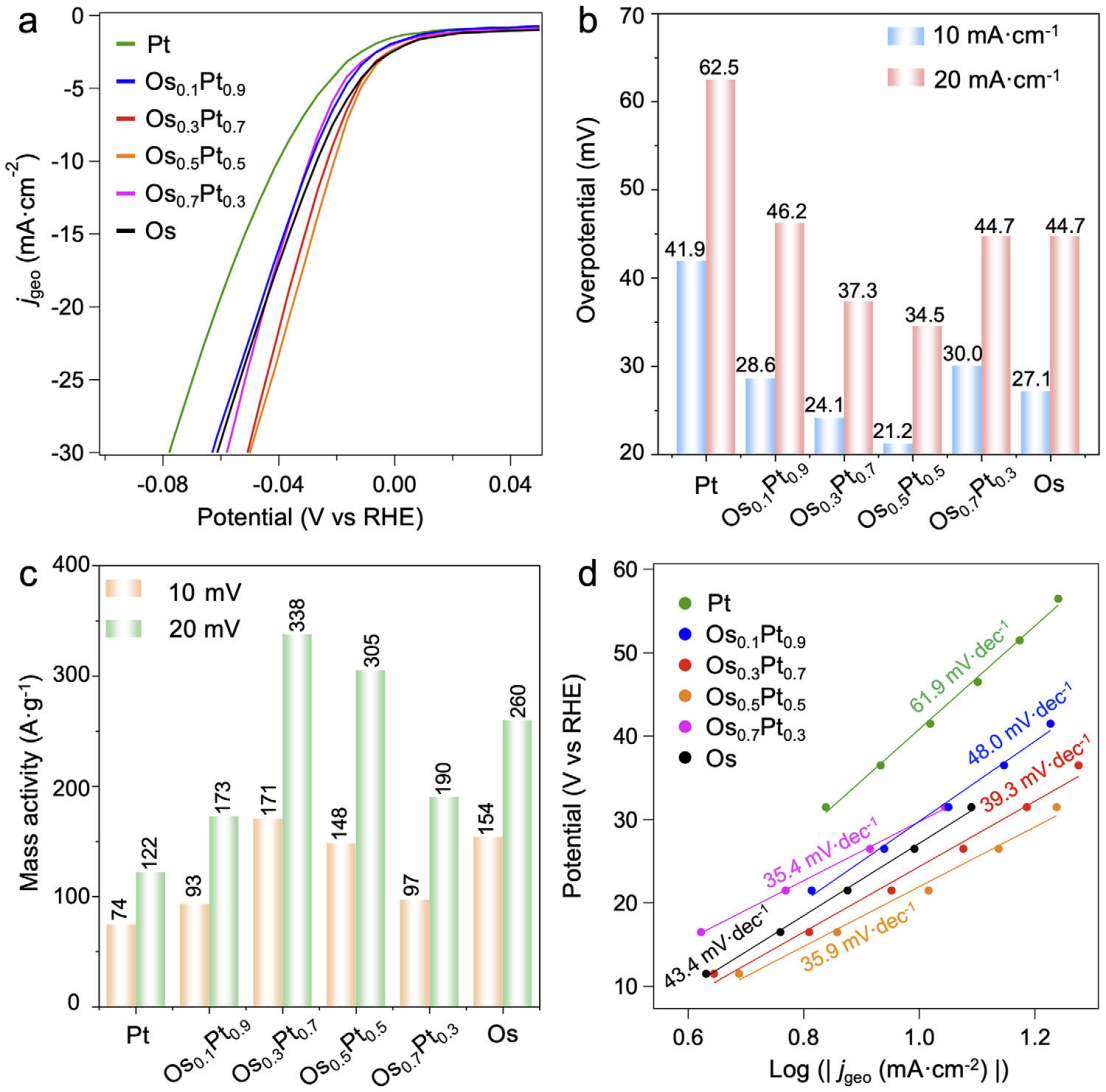
HER activities of Os_x_Pt_1‐x_/C in 1 m KOH. a) Polarization curves. b) Overpotentials at 10 mA·cm^−2^ (*η*_10_) and 20 mA·cm^−2^ (*η*_20_). c) Mass activities at overpotentials of 10 and 20 mV. d) Tafel plots.

The alkaline HER catalytic activities of Os_x_Pt_1‐x_/C in 1.0 m KOH were also investigated. All Os_x_Pt_1‐x_/C catalysts showed lower *η*_10_ and *η*_20_ than those of commercial Pt/C and Pt/C by polyol method in alkaline conditions (**Figure** **6**a,b; Figure S30, Supporting Information). Os_0.3_Pt_0.7_/C had the highest mass activity as 171 and 338 A·g_metal_^−1^ at 10 and 20 mV overpotentials, respectively (Figure 6c). After normalizing by the proportion of surface atoms, Os_0.3_Pt_0.7_/C still displayed the highest surface mass activity as 1444 A·g_surface_^−1^ at 20 mV overpotential, almost twice that of Pt/C (791 A·g_surface_^−1^) (Figure S31, Supporting Information). The lower Tafel slops of Os_x_Pt_1‐x_/C compared to Pt/C further indicated the faster kinetics, contributing to their higher HER activities (Figure 6d). Additionally, the ECSA of Os_0.3_Pt_0.7_/C and Pt/C were very similar, suggesting the higher specific area activity for Os_0.3_Pt_0.7_/C (Figures S32–S34, Supporting Information). The *R*_ct_ of Os_0.3_Pt_0.7_/C (21.6 Ω) was much smaller than that of Pt/C (63.2 Ω), further confirming the enhanced activity of Os_0.3_Pt_0.7_/C (Figure S35, Supporting Information). Os_0.5_Pt_0.5_/C also maintained higher activity than that of Pt/C after 20 h (Figure S36, Supporting Information). In addition, compared with Pt and Os‐Pt alloys, pure Os had relatively good HER activity in alkaline conditions but relatively bad activity under acid one. This might be due to the accelerated H_2_O dissociation (rate‐determining step) from strong O affinity for pure Os. The strong H_2_O dissociation for Os sites could be a novel design strategy for alkaline HER catalysts.

### 2.5 The Electronic States Engineering and its Influence on Reaction Pathway

**Figure 7:**
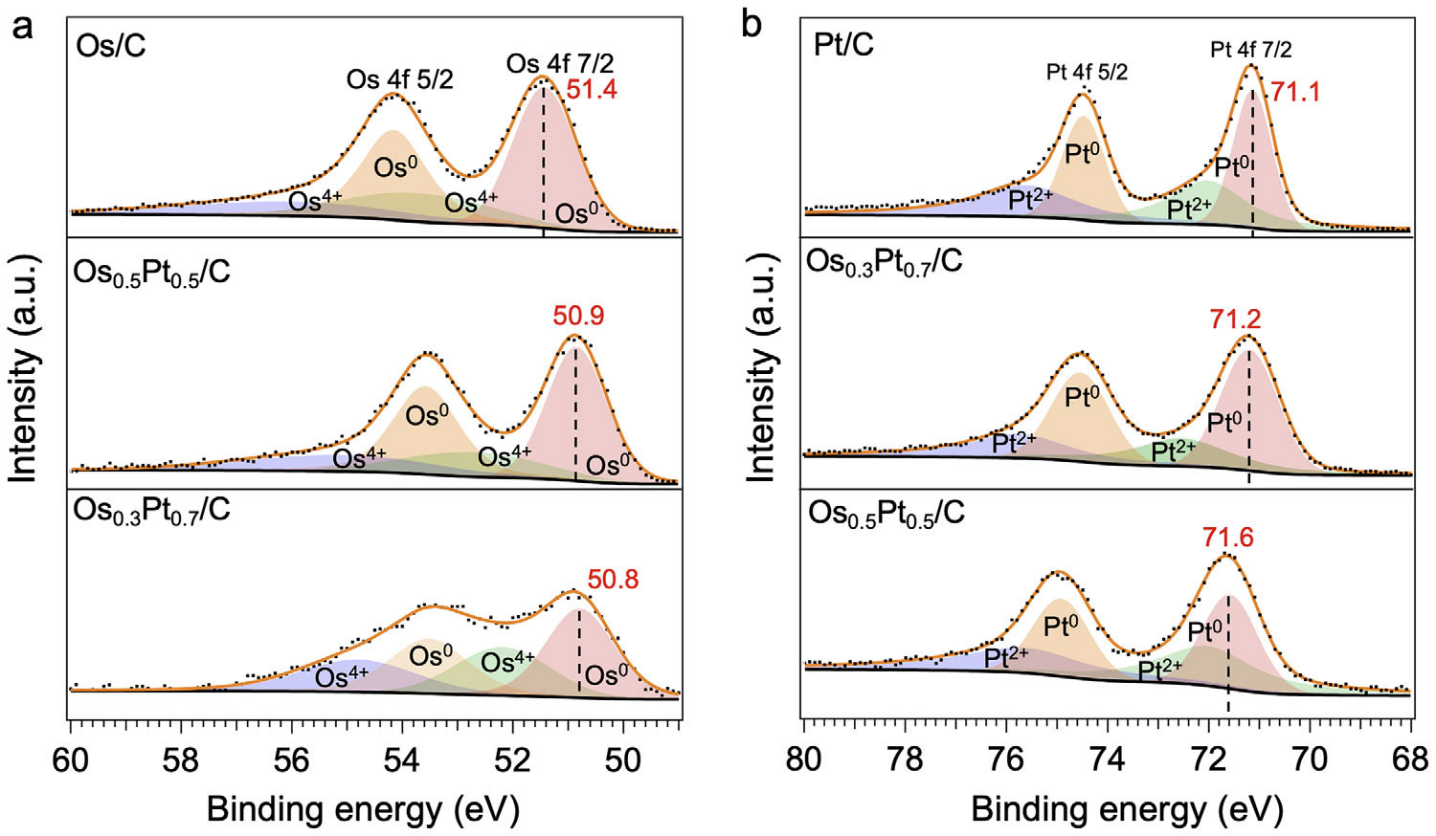
XPS spectra. XPS spectra of a) Os 4f and b) Pt 4f for Os/C, Pt/C, Os_0.5_Pt_0.5_/C, and Os_0.3_Pt_0.7_/C. The black dots and orange curves show raw data and fitting curves, respectively. The dashed lines show the locations of Os^0^ 4f 7/2 peaks (a) and Pt^0^ 4f 7/2 peaks (b).

To investigate the electronic states of Os_x_Pt_1‐x_/C, X‐ray photoelectron spectroscopic (XPS) measurements were carried out for Os_0.3_Pt_0.7_/C and Os_0.5_Pt_0.5_/C samples with Os/C and Pt/C as references (Table S9, Supporting Information). As shown in **Figure** **7**a, the binding energy of the Os^0^‐4f orbital decreased as Os content decreased, indicating electron enrichment on Os in Os_x_Pt_1‐x_/C. The Pt^0^‐4f peaks right shifted with increasing Pt content, confirming the electron transfer from Pt to Os in Os_x_Pt_1‐x_/C (Figure 7b), which configured the new electronic states and induced the corresponding new chemical properties different from pure Pt and Os.

**Figure 8:**
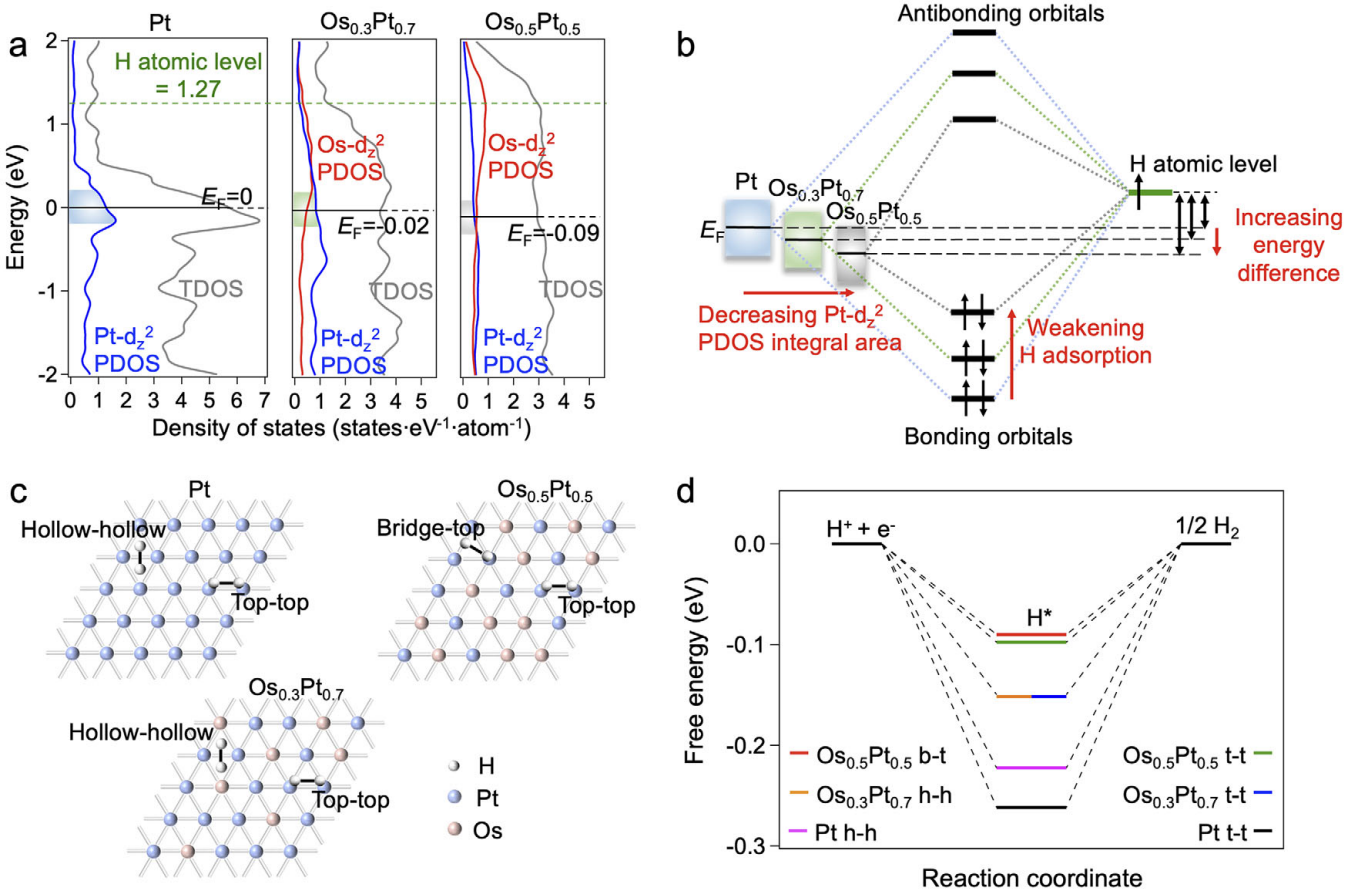
HER mechanism analysis. a) DOS of the Pt (111), fcc‐Os_0.5_Pt_0.5_ (111), and fcc‐Os_0.3_Pt_0.7_ (111). The gray, red, and blue curves show the total DOS (TDOS), Os‐d_z_^2^ partial DOS, and Pt‐d_z_^2^ partial DOS, respectively. The black and green dashed lines show the Fermi level of each sample and the H atomic level, respectively. b) The bonding and antibonding orbitals formations between H atom and Pt‐d_z_^2^ orbitals in Pt, fcc‐Os_0.5_Pt_0.5_, and fcc‐Os_0.3_Pt_0.7_. The energy difference increasing and Pt‐d_z_^2^ partial DOS integral area decreasing induced the H adsorption weakening. c) Possible H_2_ desorption couple sites on Pt (111), fcc‐Os_0.5_Pt_0.5_ (111), and fcc‐Os_0.3_Pt_0.7_ (111) surfaces. d) Diagram of the average H adsorption energy of most favorable couple sites on Pt (111), fcc‐Os_0.5_Pt_0.5_ (111) and fcc‐Os_0.3_Pt_0.7_ (111) surfaces.

To understand the influence of electronic states engineering on HER catalytic activities, DFT calculations were performed for Pt (111), fcc‐Os_0.3_Pt_0.7_ (111), and fcc‐Os_0.5_Pt_0.5_ (111) slabs (Figure S37, Supporting Information). The density of states (DOS) calculations showed that the Fermi levels (*E*_F_) of Os_x_Pt_1‐x_ decreased as the Pt content decreased (**Figures** **8**a and S38, Supporting Information). For Pt, fcc‐Os_0.3_Pt_0.7_, and fcc‐Os_0.5_Pt_0.5_, the energy difference between H atomic level and *E*_F_ increased with decreasing Pt content, leading to higher Pt─H bonding orbital energy and weaker H─Pt adsorption (Figure 8b).^[^ ^26^ ^]^ On the other hand, the integral areas of Pt‐d_z_^2^ partial DOS (PDOS) near *E*_F_ decreased as the Pt content decreased, also contributing to higher Pt─H bonding orbital energy and weaker H─Pt adsorption (Figure 8b).^[^ ^26^ ^]^ Therefore, the weakest H─Pt adsorption would be obtained for Os_0.5_Pt_0.5_ among Pt, fcc‐Os_0.3_Pt_0.7_, and fcc‐Os_0.5_Pt_0.5_.

To verify the influence of electronic states on H adsorption abilities, the Gibbs free energies of hydrogen adsorption (*ΔG*_H_) on the top, bridge, and hollow sites were calculated for Pt (111), fcc‐Os_0.3_Pt_0.7_ (111), and fcc‐Os_0.5_Pt_0.5_ (111) (Figures S39‐S41; Table S10, Supporting Information). Considering H_2_ desorbs from two adjacent H adsorption sites, the average *ΔG*_H_ of the coupled sites would determine the H_2_ desorption processes.^[^ ^27^ ^]^ Within the feasible geometrical distance between coupled sites, we compared the average *ΔG*_H_ of possible H_2_ generation sites, including hollow‐hollow (h‐h), hollow‐top (h‐t), bridge‐top (b‐t), and top‐top (t‐t) sites for Pt, fcc‐Os_0.3_Pt_0.7_, and fcc‐Os_0.5_Pt_0.5_ (Figure 8c; Table S11, Supporting Information). As the most favorable H_2_ desorption couple sites for fcc‐Os_0.3_Pt_0.7_, the t‐t and h‐h sites were found with average *ΔG*_H_ as both −0.15 eV, closer to zero compared with pure Pt (−0.22 and −0.26 eV at h‐h and t‐t sites, respectively), proving more efficient H_2_ desorption processes (Figure 8d). While the b‐t and t‐t sites were the most energy‐favored for fcc‐Os_0.5_Pt_0.5_, with average *ΔG*_H_ values of −0.09 and −0.10 eV, respectively (Figure 8d), these values are much closer to zero compared to those of pure Pt and fcc‐Os_0.3_Pt_0.7_. The close‐to‐zero *ΔG*_H_ for fcc‐Os_0.5_Pt_0.5_ and fcc‐Os_0.3_Pt_0.7_ were achieved by electronic state modification of Pt sites from Os solid‐solution alloying. Therefore, the reaction pathways, including H_2_ desorption, were optimized with lower energy barriers for fcc‐Os_0.3_Pt_0.7_ and fcc‐Os_0.5_Pt_0.5_ in comparison to Pt. Considering the larger number of more active Pt sites in fcc‐Os_0.3_Pt_0.7_ relative to fcc‐Os_0.5_Pt_0.5_, the overall HER activity of fcc‐Os_0.3_Pt_0.7_ surpassed that of fcc‐Os_0.5_Pt_0.5_. Based on the above discussions, we demonstrated that by extensively adjusting the electronic states through solid‐solution alloying of Os and Pt, the HER reaction pathways could be essentially modified to obtain superior catalytic activities, significantly enhanced compared to those of pure Pt.

## 3 Conclusion

In summary, we proposed a one‐step in situ polyol method realizing steady‐state synthesis and phase control of immiscible Os_x_Pt_1‐x_/C catalysts, with homogeneous distribution of Os and Pt elements and single dispersion of fcc‐Os_x_Pt_1‐x_ NPs on support. Most fcc‐Os_x_Pt_1‐x_/C catalysts exhibited superior HER catalytic activities in both acid and alkaline conditions compared to pure Pt/C. The *η*_10_ of fcc‐Os_0.3_Pt_0.7_/C in 0.5 m H_2_SO_4_ was only 1.0 mV as the top‐level HER catalyst. According to DFT calculations, the decreased Pt‐d_z_^2^ PDOS integral area and lowered *E*_F_ resulted in weaker H‐Pt adsorption for fcc‐Os_0.3_Pt_0.7_ and fcc‐Os_0.5_Pt_0.5_. The closer‐to‐zero *ΔG*_H_ of fcc‐Os_0.3_Pt_0.7_ and fcc‐Os_0.5_Pt_0.5_ demonstrated more efficient H_2_ desorption processes undergoing the modified HER pathways, intrinsically determined by tunning the electronic states through atomic‐level mixing Os to Pt. The electronic‐state engineering strategy using solid‐solution alloys in immiscible systems could significantly inspire the development of novel functional materials with outstanding performance. The established steady‐state one‐step in situ methods may open a new avenue for facile preparations of supported materials with binary/ternary solid‐solution types and high‐entropy alloys or compounds including immiscible systems.

## Conflict of Interest

The authors declare no conflict of interest.

## Supporting information



Supporting Information

## Data Availability

The data that support the findings of this study are available in the supplementary material of this article.
