# Supplementary material for: One‐Step Syntheses of Face‐Centered Cubic OsxPt1‐x/C with Near‐Zero‐Overpotential Hydrogen Evolution from Electronic‐State Engineering
Source: Adv Sci (Weinh). 2025 May 11;12(28):2504161. doi: 10.1002/advs.202504161 (PMC12302605; doi:10.1002/advs.202504161)
Supplement: Supplementary file 1 — Supporting Information [file ADVS-12-2504161-s001.docx]

**Supporting Information**

**One-Step Syntheses of Face-Centered Cubic Os_x_Pt_1-x_/C with Near-Zero-Overpotential Hydrogen Evolution from Electronic-State Engineering**

Junyun Gao,^1,2^ and Bo Huang^1,2,3,4,*^

^1^School of Chemical Engineering and Technology, Xi’an Jiaotong University, Western China Science and Technology Innovation Harbor, Xixian-ward, Xi’an 712000, China.

^2^Shaanxi Tianyi Element Technology Co., Ltd, High-tech industrial development zone, Xianyang 712000, China.

^3^School of Future Technology, Xi’an Jiaotong University, Western China Science and Technology Innovation Harbor, Xixian-ward, Xi’an 712000, China

^4^National Innovation Platform (Center) for Industry-Education Integration of Energy Storage Technology, Xi’an Jiaotong University. Western China Science and Technology Innovation Harbor, Xixian-ward, Xi’an 712000, China.

*Correspondence and requests for materials should be addressed to B. Huang (email: bohuang@xjtu.edu.cn).

**1. Experimental section/Methods**

*Chemicals.* Potassium hexachloroosmate (IV) (K_2_OsCl_6_, Os purity ≥ 38.7%) was purchased from Haohong Biopharmaceutical Technology Co., Ltd. Chloroplatinic acid (H_2_PtCl_6_·6H_2_O, Pt purity ≥ 37.5%) was purchased from Shanghai Macklin Biochemical Co., Ltd. Diethylene glycol (DEG, 99%) was purchased from Meryer. Commercial Pt/C (Premetek 10% Pt/ Vulcan XC-72) was purchased from SCIMaterials Hub. Polyvinylpyrrolidone (PVP) was purchased from Energy Chemical. Ethyl acetate (EtOAc, 98.5%) was purchased from Chemical Reagent. Nafion (5%) was purchased from Canrd. Isopropanol (C_3_H_8_O. 99.7%) was purchased from Xilong Scientific. Sodium borohydride (NaBH_4_, 98% purity) was purchased from Damao Chemical Regent Factory. Deionized water (H_2_O) was prepared from LPD-I-10T Ultra-pure water system.

*Syntheses of Os_x_Pt_1-x_/C catalysts.* The Os_x_Pt_1-x_/C catalysts were prepared by one-step polyol method. Taking Os_0.1_Pt_0.9_/C as example, solution A was DEG (110 ml) containing acetylene black carbon (256.8 mg). The solution A was degassing by liquid N_2_ three times. K_2_O_s_Cl_6_ (11.5 mg) and H_2_PtCl_6_·6H_2_O (63.2 mg) were dissolved in DEG (5 ml) and H_2_O (2 ml) as solution B. Then solution B was dropwise added into solution A within 2 min at 230 °C under N_2_ flow. The black solution was kept at 230 °C for another 5 min to guarantee the complete reduction. After cooling to room temperature, the black solution was washed with ethyl acetate three times and centrifugated. The obtained black powder was stored under vacuum condition. Other Os_x_Pt_1-x_/C catalysts (x=0, 0.3, 0.5, 0.7, 1) were prepared by controlling the molar ratio of precursors and the details were shown in Table S1.

*Synthesis of Pt/C by polyol method.* Acetylene black carbon (175.6 mg) was added into DEG (100 ml) containing H_2_PtCl_6_·6H_2_O (51.8 mg). After stirring for 4 h, the solution was heated to 230 °C and kept for another 5 min. The dark solution was washed twice with ethyl acetate. The black powder was obtained after centrifugation and vacuum drying.

*Characterizations.* The crystal structures were investigated by XRPD analysis using a Shimadzu XRD-6100 diffractometer (Cu *K*𝛼 radiation). The atomic ratios of Os and Pt in the alloys were investigated by XRF and EDX spectroscopies using BXR 616 and Talos F200X, respectively. HAADF-STEM and STEM-EDX mapping analyses were recorded on Talos F200X operated at 200 kV accelerating voltage. XPS spectra for samples on a carbon sheet were investigated by Thermo Fisher Scientific ESCALAB Xi+ X-ray photoelectron spectrometer without surface etching treatment. The binding energies were corrected with the reference to the C(1s) line at 284.3 eV of conductive carbon shape.

*HER catalytic performance measurements.* The HER activities of fcc-Os_x_Pt_1-x_/C were investigated using three-electrode system and all of the electrochemical data were collected with a CHI 650E electrochemical analyzer (CH Instruments). Catalyst-coated glassy carbon electrode, graphite rod and Hg/Hg_2_SO_4_ electrode in 0.5 M H_2_SO_4_ (or Hg/HgO electrode in 1.0 M KOH) were used as work electrode, counter electrode and reference electrode, respectively. The LSV curves were collected with iR-compensated. For preparing catalyst suspension (including commercial Pt/C), catalyst (5 mg) were added into the solution including isopropanol (1300 μL), deionized H_2_O (600 μL) and Nafion solution (100 μL, 5 wt%). The suspension was then sonicated for 4 h to obtain better dissolution. The work electrode was prepared by dropping catalyst suspension (10 μL) on the glassy carbon electrode.

*Density functional theory (DFT) calculation.* We used the DFT as implemented in the Vienna Ab initio simulation package (VASP) in all calculations.^[1]^ The exchange-correlation potential is described by using the generalized gradient approximation of Perdew-Burke-Ernzerhof (GGA-PBE).^[2]^ The projector augmented-wave (PAW) method is employed to treat interactions between ion cores and valence electrons. The plane-wave cutoff energy was fixed to 450 eV. Given structural models were relaxed until the Hellmann–Feynman forces smaller than -0.03eV/Å and the change in energy smaller than 10^-4^ eV was attained.

For the top layer DOS calculation, Grimme’s DFT-D3 methodology was used to describe the dispersion interactions among all the atoms in adsorption models. When the same vacuum layer thickness was fixed (12 Å), we got the calculated Fermi level of Pt (111), fcc-Os_0.5_Pt_0.5_ (111) and fcc-Os_0.3_Pt_0.7_ (111) as 4.13, 3.72, and 4.04 eV, respectively. The work functions of Os, Pt were reported as 5.93 and 5.65 eV,^[3]^ respectively, which were used to calibrate the Fermi levels. To compare the energy level of the metal slabs and H_2_ orbitals, we set the *E*_F_ of Pt as 0 eV, and obtained the calibrated *E*_F_ for fcc-Os_0.5_Pt_0.5_ (111) and fcc-Os_0.3_Pt_0.7_ (111) as –0.09 and -0.02, respectively.

**Table S1.** Experimental details for the syntheses of Os_x_Pt_1-x_/C.

| Samples | K_2_O_s_Cl_6_  （mg） | H_2_PtCl_6_·6H_2_O （mg） | Acetylene black carbon  （mg） | DEG  （ml） |
| --- | --- | --- | --- | --- |
| Os_0.1_Pt_0.9_/C | 11.5 | 63.2 | 256.8 | 110 |
| Os_0.3_Pt_0.7_/C | 9.2 | 12.9 | 77.1 | 80 |
| Os_0.5_Pt_0.5_/C | 42.3 | 25.9 | 238.4 | 110 |
| Os_0.7_Pt_0.3_/C | 59.7 | 15.5 | 264.9 | 110 |
| Os/C | 72.2 | 0 | 256.8 | 110 |

**Table S2.** Atomic ratios of Os_x_Pt_1-x_/Al_2_O_3_ by XRF.

| Samples | Os at% | Pt at% |
| --- | --- | --- |
| Os_0.1_Pt_0.9_ | 15.3 | 84.7 |
| Os_0.3_Pt_0.7_ | 29.6 | 70.4 |
| Os_0.5_Pt_0.5_ | 56.7 | 43.3 |
| Os_0.7_Pt_0.3_ | 65.5 | 34.5 |

**2. Characterizations.**

**2.1 XRD patterns.**


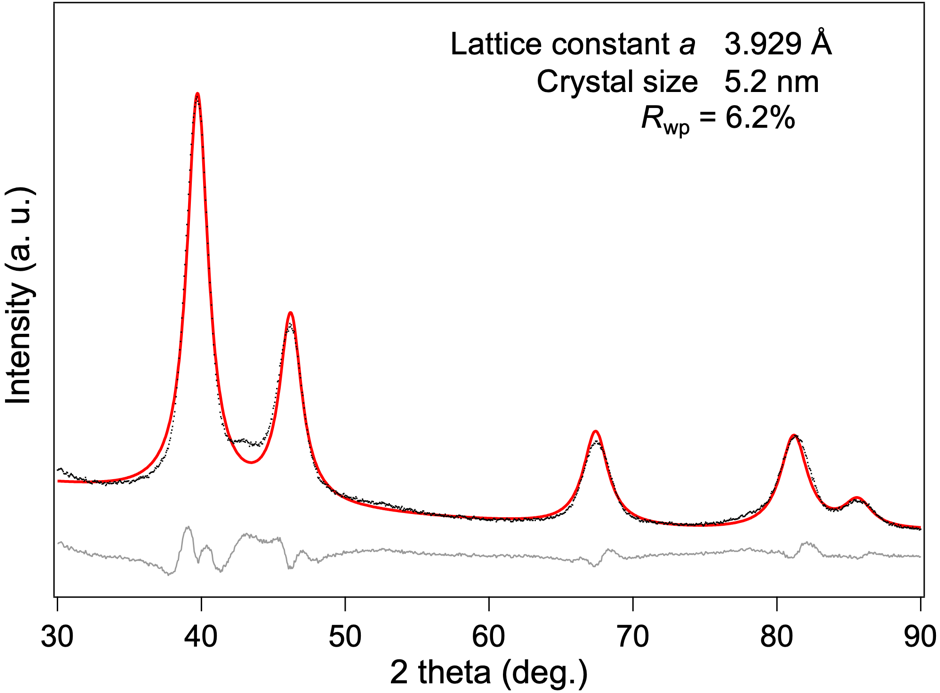


**Figure S1.** XRPD pattern of Pt/C catalyst (black dots) at 303 K and calculated pattern (red line). The bottom lines show the difference profile (gray). The radiation wavelength was 1.54056 Å.


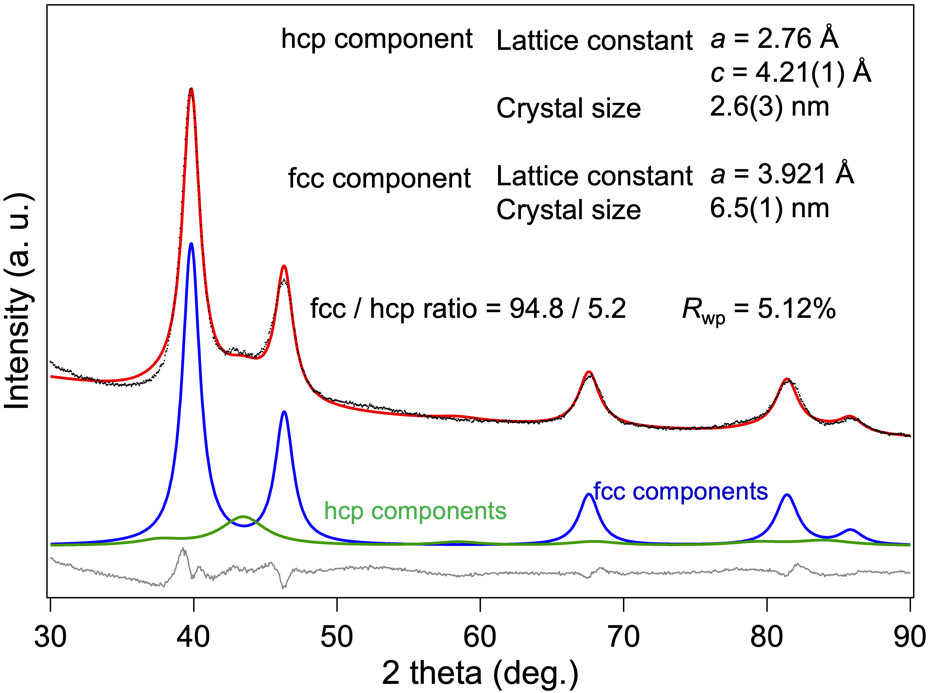


**Figure S2.** XRPD pattern of Os_0.1_Pt_0.9_/C catalyst (black dots) at 303 K and calculated pattern (red line). The bottom lines show the difference profile (gray). The radiation wavelength was 1.54056 Å.


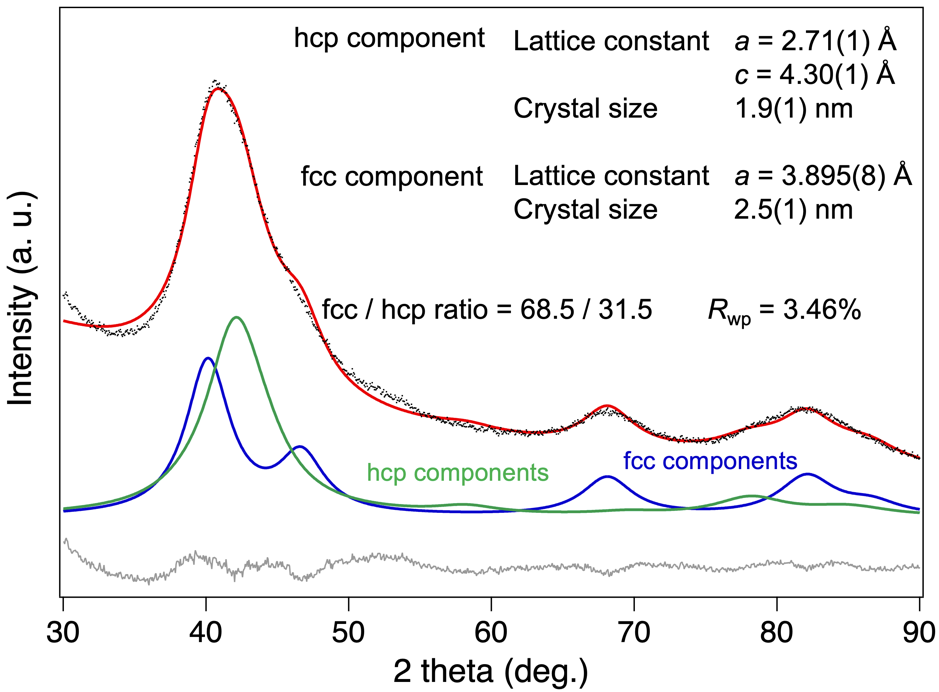


**Figure S3.** XRPD pattern of Os_0.5_Pt_0.5_/C catalyst (black dots) at 303 K and calculated pattern (red line). The bottom lines show the difference profile (gray). The radiation wavelength was 1.54056 Å.


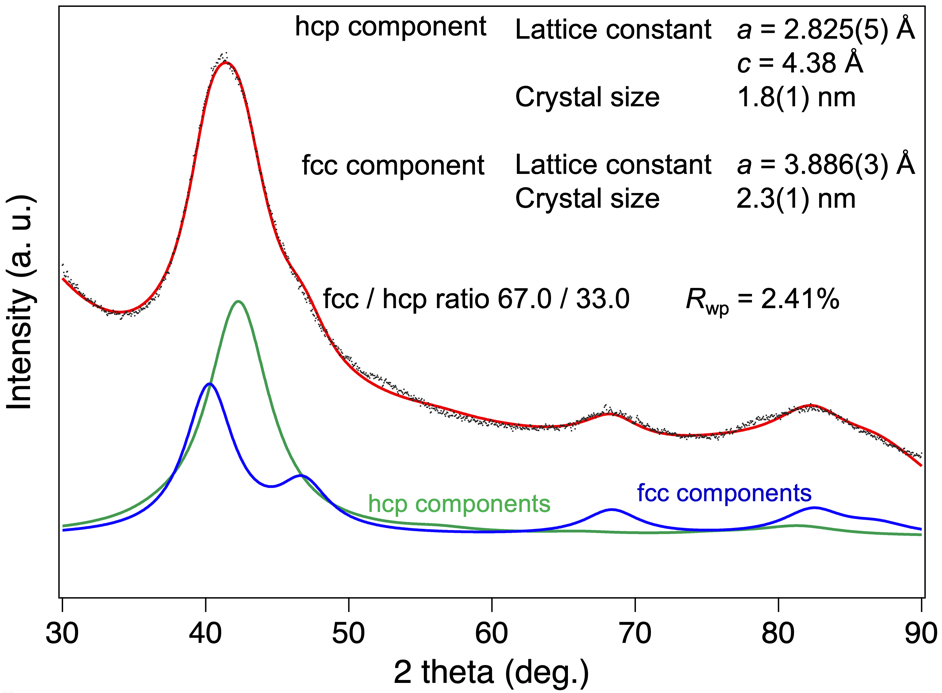


**Figure S4.** XRPD pattern of Os_0.7_Pt_0.3_/C catalyst (black dots) at 303 K and calculated pattern (red line). The bottom lines show the difference profile (gray). The radiation wavelength was 1.54056 Å.


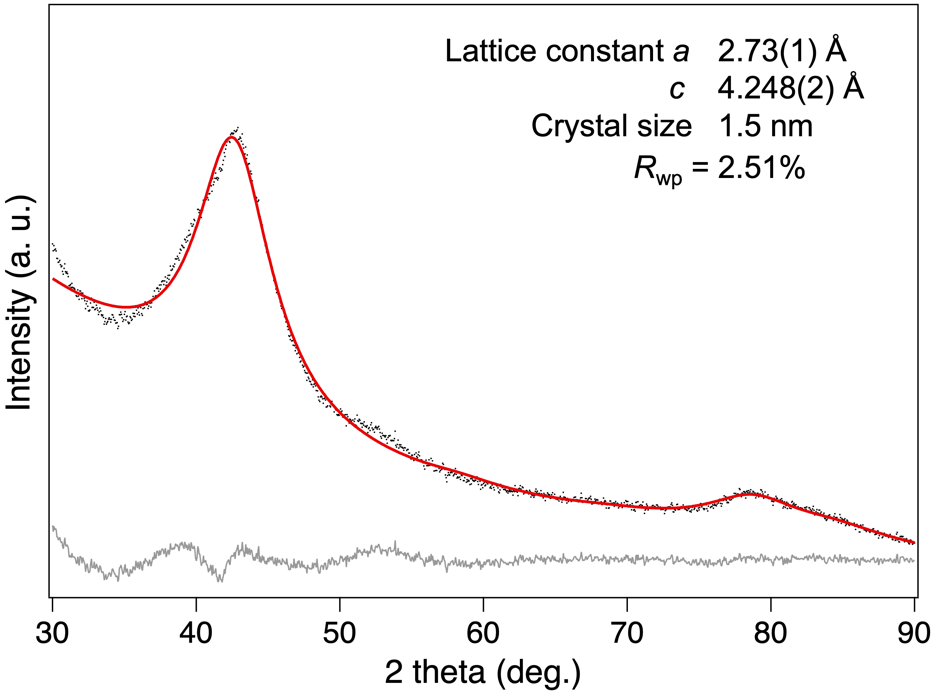


**Figure S5.** XRPD pattern of Os/C catalyst (black dots) at 303 K and calculated pattern (red line). The bottom lines show the difference profile (gray). The radiation wavelength was 1.54056 Å.

**2.2 HAADF-STEM image and histogram.**


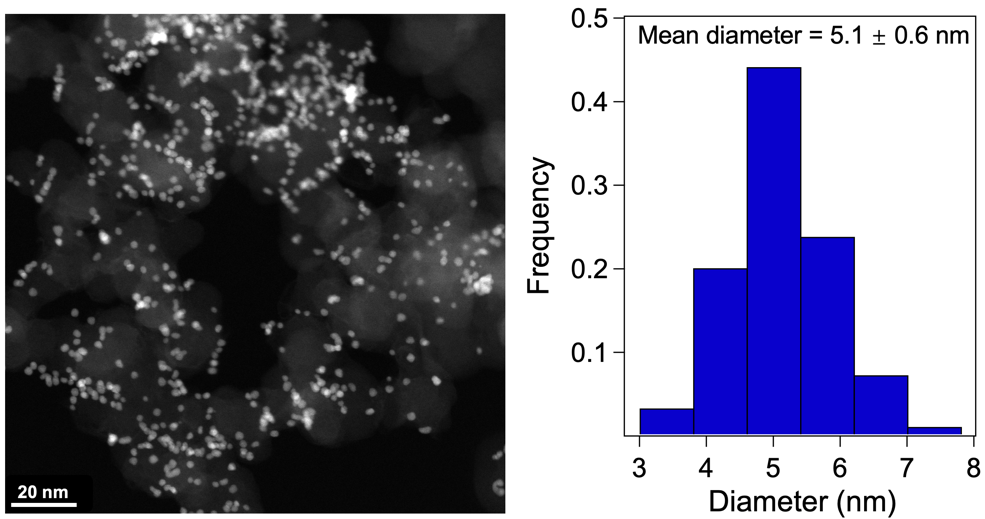


**Figure S6.** HAADF-STEM image and histogram of Pt NPs.


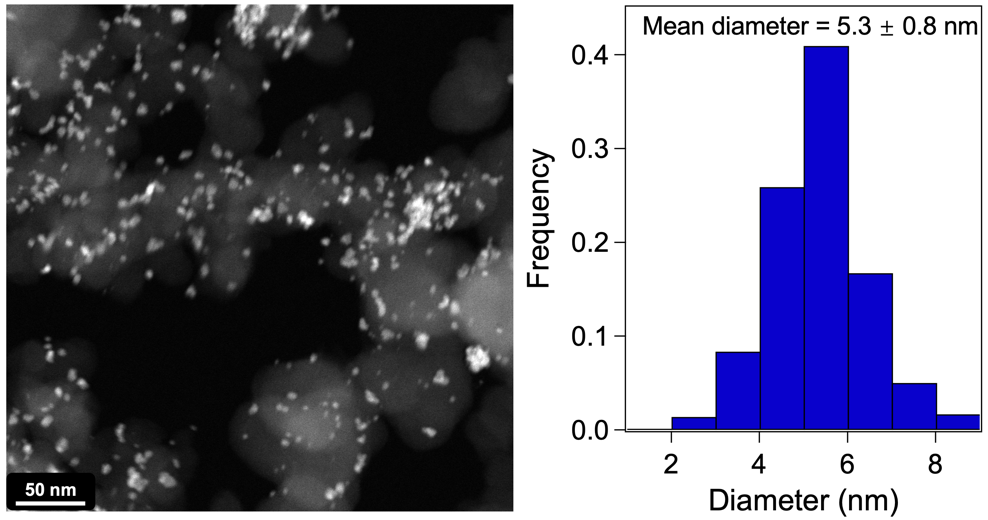


**Figure S7.** HAADF-STEM image and histogram of Os_0.1_Pt_0.9_ NPs.


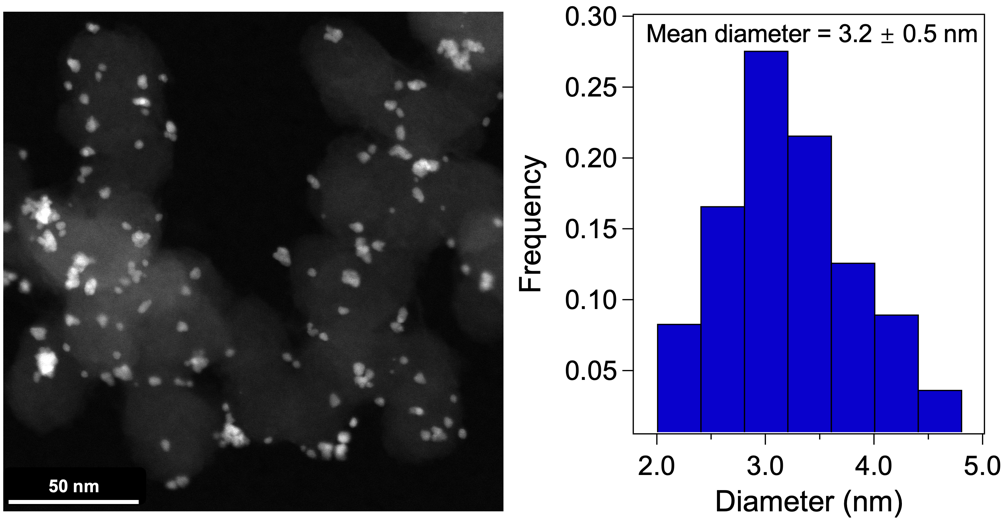


**Figure S8.** HAADF-STEM image and histogram of Os_0.3_Pt_0.7_ NPs.


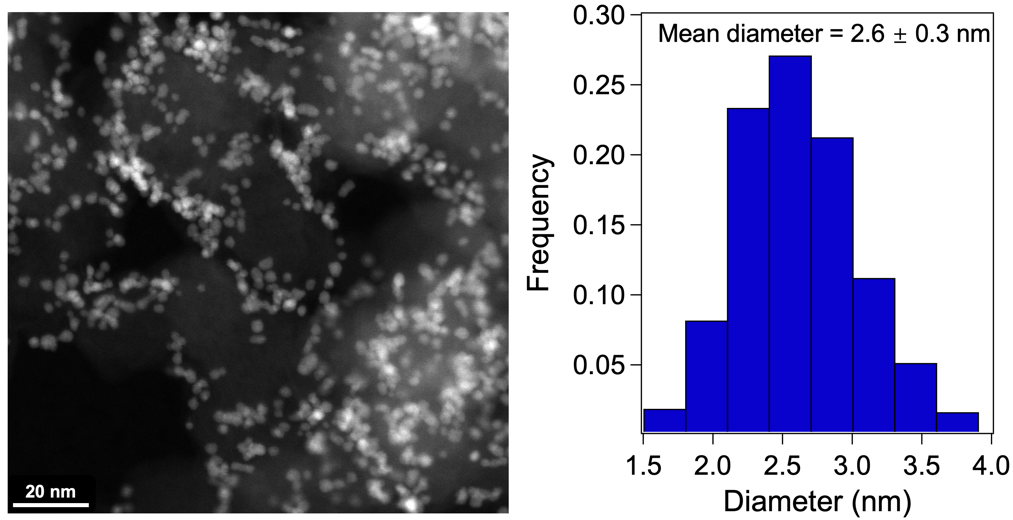


**Figure S9.** HAADF-STEM image and histogram of Os_0.5_Pt_0.5_ NPs.


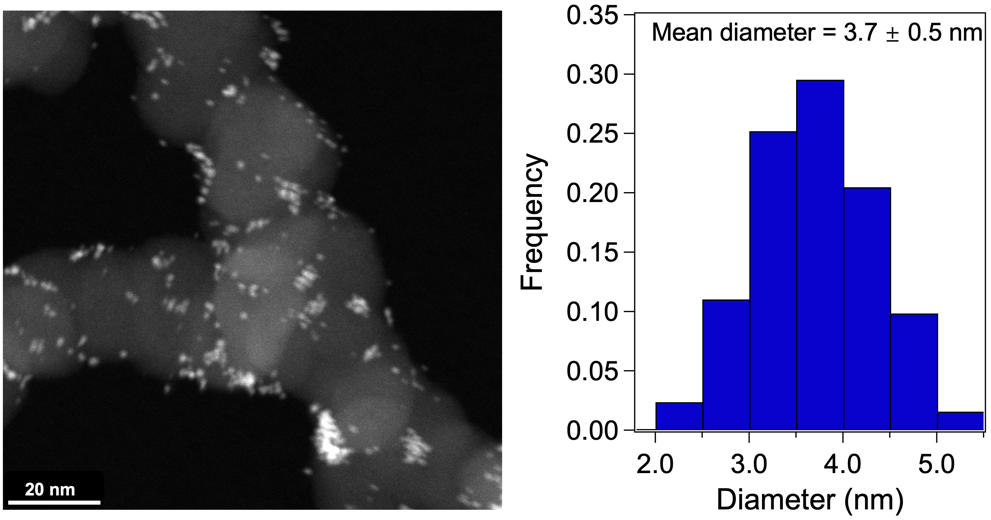


**Figure S10.** HAADF-STEM image and histogram of Os_0.7_Pt_0.3_ NPs.


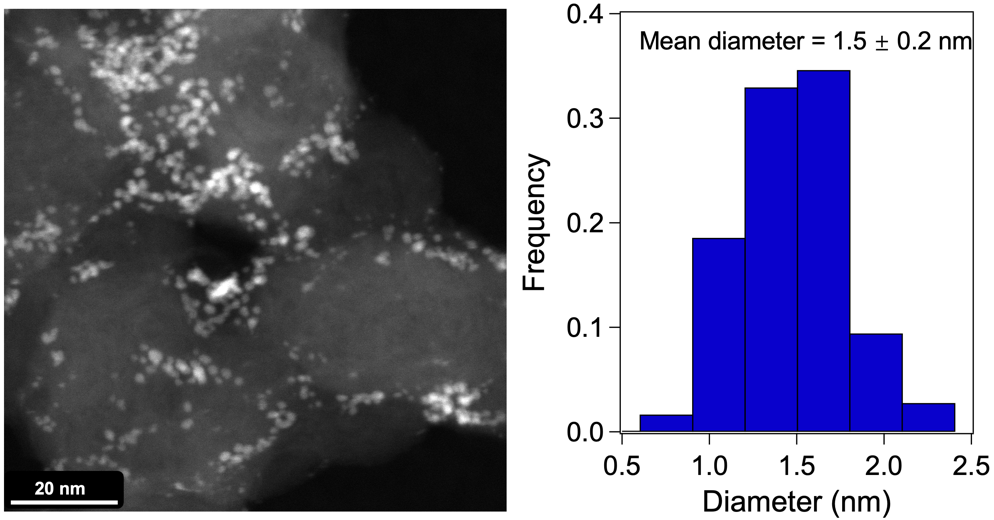


**Figure S11.** HAADF-STEM image and histogram of Os_0.9_Pt_0.1_ NPs.


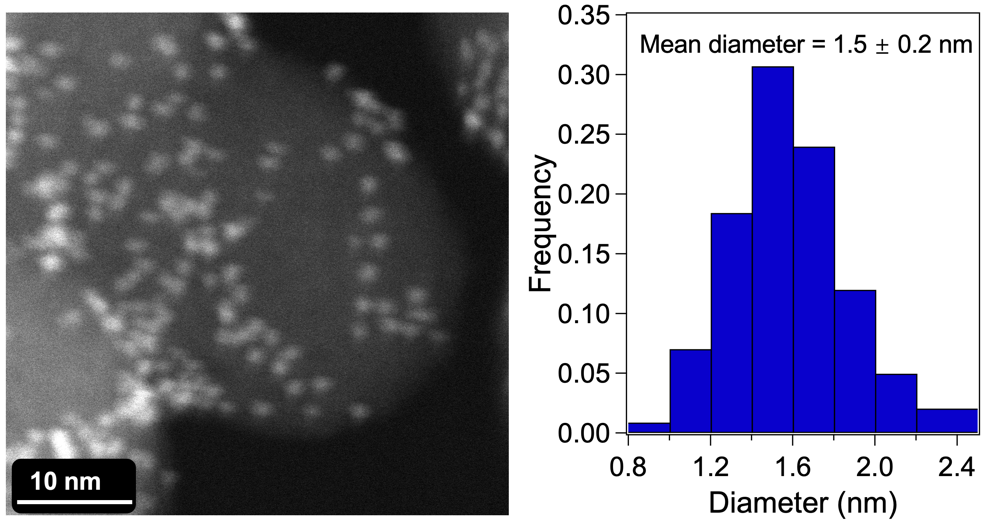


**Figure S12.** HAADF-STEM image and histogram of Os NPs.

**2.3 STEM-EDX images and compositions determined by EDX and XRF.**


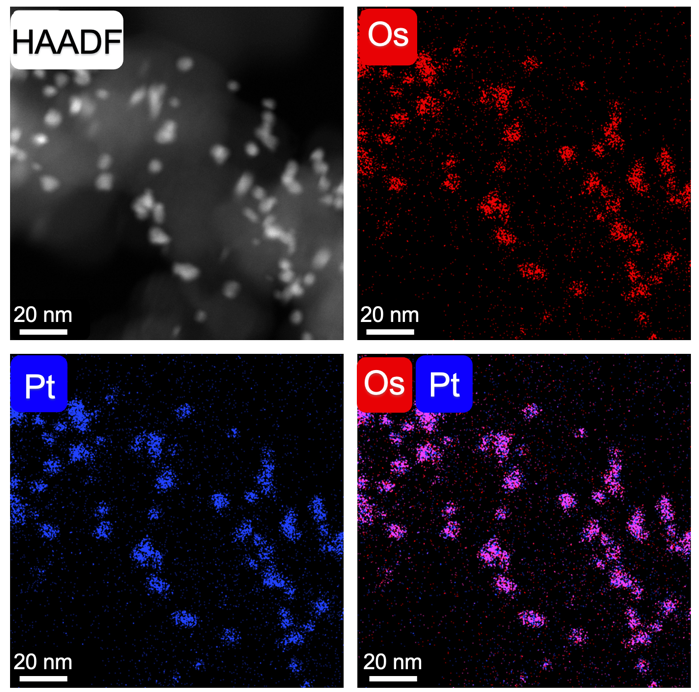


**Figure S13.** HAADF-STEM image, Os–L STEM-EDX map (red), Pt–L STEM-EDX map (blue), and an overlay map of the Os and Pt distributions obtained from the Os_0.1_Pt_0.9_/C NPs.


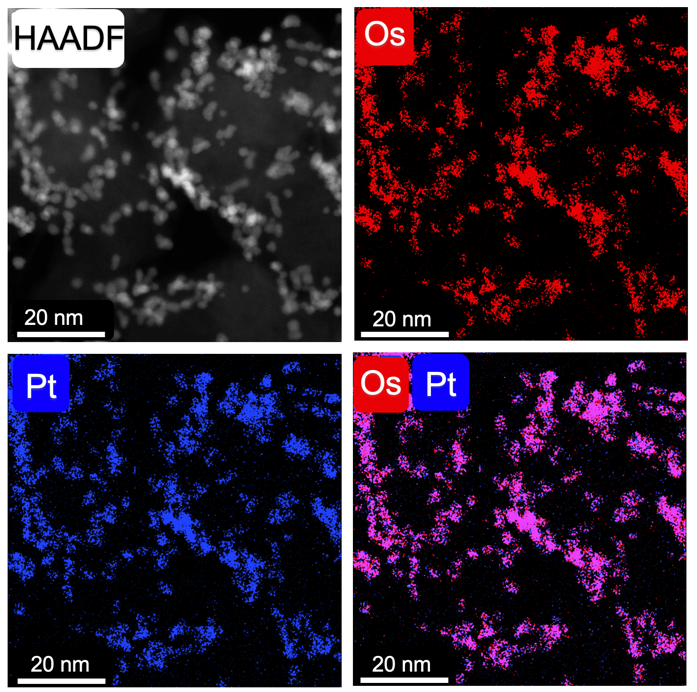


**Figure S14.** HAADF-STEM image, Os–L STEM-EDX map (red), Pt–L STEM-EDX map (blue), and an overlay map of the Os and Pt distributions obtained from the Os_0.5_Pt_0.5_/C NPs.


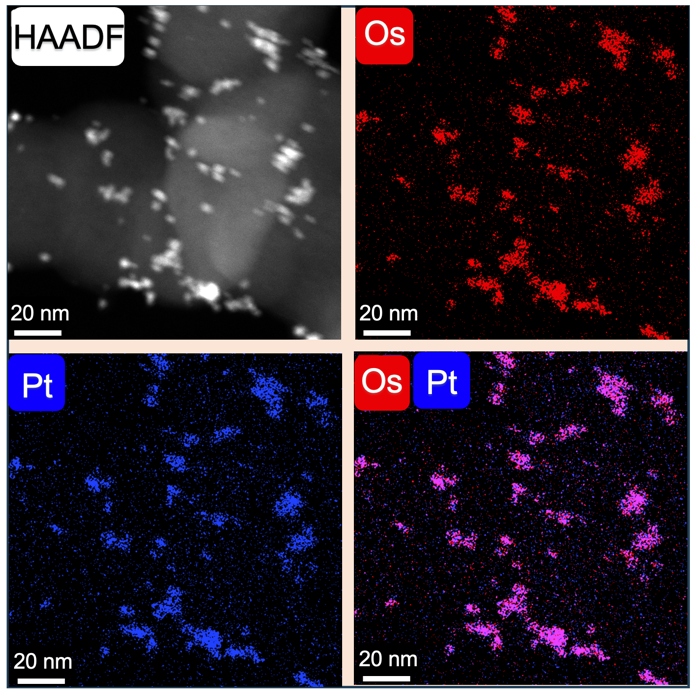


**Figure S15.** HAADF-STEM image, Os–L STEM-EDX map (red), Pt–L STEM-EDX map (blue), and an overlay map of the Os and Pt distributions obtained from the Os_0.7_Pt_0.3_/C NPs.


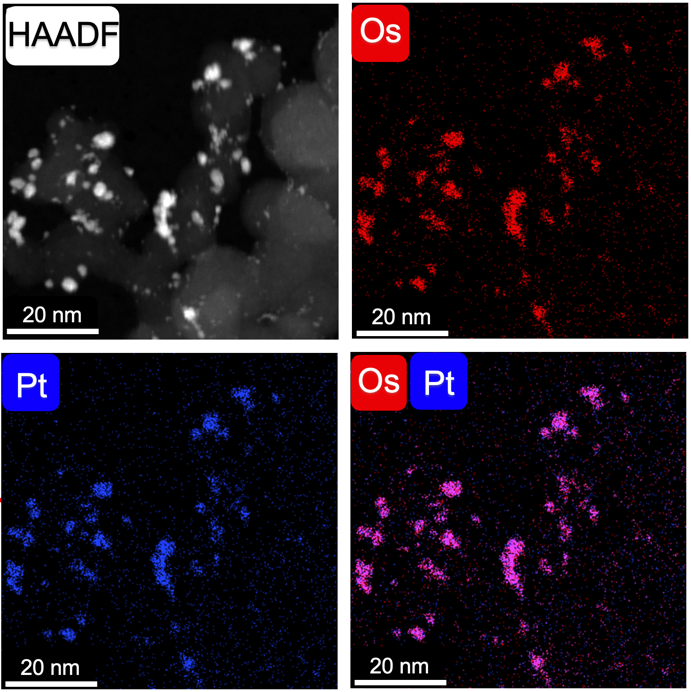


**Figure S16.** HAADF-STEM image, Os–L STEM-EDX map (red), Pt–L STEM-EDX map (blue), and an overlay map of the Os and Pt distributions obtained from the Os_0.9_Pt_0.1_/C NPs.

**Table S3.** Atomic ratios of Os_x_Pt_1-x_/Al_2_O_3_ determined by EDX-STEM measurements.

| Samples | Os at% | Pt at% |
| --- | --- | --- |
| Os_0.1_Pt_0.9_ | 13.3 | 86.7 |
| Os_0.3_Pt_0.7_ | 32.3 | 67.7 |
| Os_0.5_Pt_0.5_ | 47.8 | 52.2 |
| Os_0.7_Pt_0.3_ | 67.1 | 32.9 |

**Table S4.** Comparison of Os/Pt ratios in Os_x_Pt_1-x_/C evaluated by EDX mapping, XRF measurements, XRPD Rietveld refinements with nominal ratio.

| Samples | Nominal ratio of Os/Pt (%) | EDX ratio of Os/Pt (%) | XRF ratio of Os/Pt (%) | XRPD refinement ratio of Os/Pt (%) |
| --- | --- | --- | --- | --- |
| Os_0.1_Pt_0.9_ | 10/90 | 13.3/86.7 | 15.3/84.7 | 13.1/86.9 |
| Os_0.3_Pt_0.7_ | 30/70 | 32.3/67.7 | 29.6/70.4 | 26.2/73.8 |
| Os_0.5_Pt_0.5_ | 50/50 | 47.8/52.2 | 56.7/43.3 | 54.4/45.6 |
| Os_0.7_Pt_0.3_ | 70/30 | 67.1/32.9 | 65.5/34.5 | 70.0/30.0 |

**3. Synthesis and characterizations of samples for comparison.**

**3.1 Synthesis of Os_0.3_Pt_0.7_ without support with suitable reducibility (OP-DEG 230).**

The solution A (240 ml) was degassing by liquid N_2_ three times. 27.4 mg K_2_O_s_Cl_6_ and 38.8 mg H_2_PtCl_6_·6H_2_O were dissolved in 5 ml DEG and 2 ml H_2_O as solution B. Then solution B was dropwise added into solution A within 2 min at 230 °C under N_2_ flow. The black solution was kept at 230 °C for another 5 min. After cooling to room temperature, the black solution was washed with ethyl acetate three times and centrifugated. The obtained black powder was stored under vacuum condition. The XRPD pattern of OP-DEG 230 was shown in Figure S17. As shown in Figure S17, the sharp peaks location of OP-DEG 230 were close to those of simulated Pt, confirming the formation of large Pt-rich NPs.


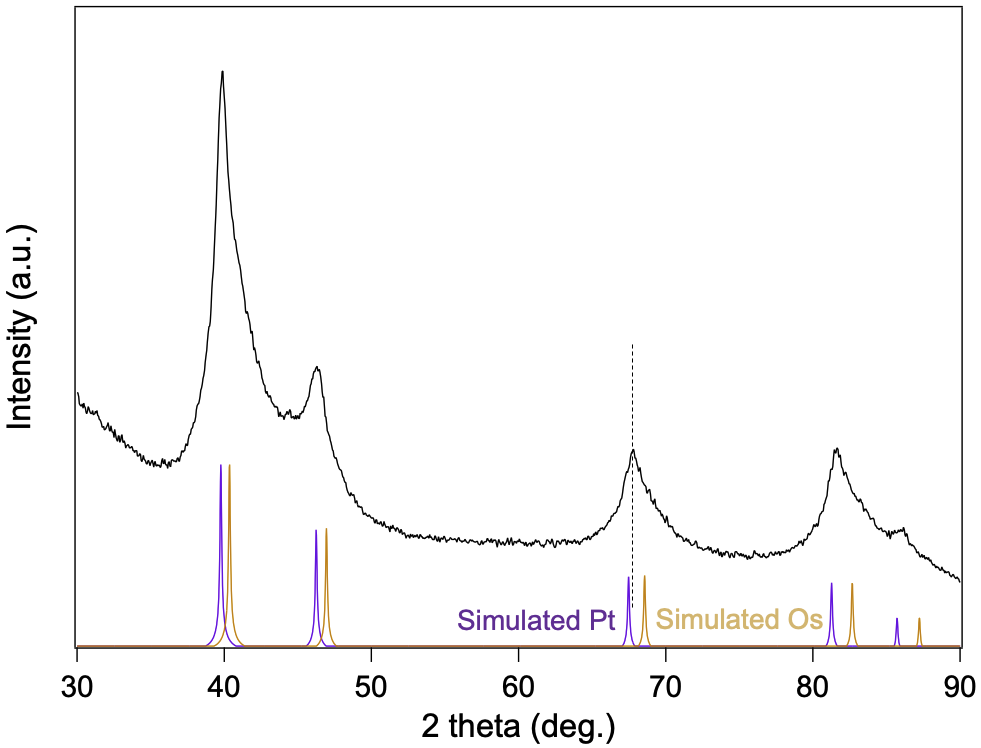


**Figure S17.** XRPD pattern of OP-DEG 230 (Os_0.3_Pt_0.7_) catalyst (black dots) at 303 K. The black curves showed the raw data. The radiation wavelength was 1.54056 Å.

**3.2 Synthesis of Os_0.3_Pt_0.7_/C (OP-180) with weak reducibility.**

The solution A was 80 ml DEG containing 77.1 mg acetylene black carbon. The solution A was degassing by liquid N_2_ three times. 9.2 mg K_2_O_s_Cl_6_ and 12.9 mg H_2_PtCl_6_·6H_2_O were dissolved in 5 ml DEG and 2 ml H_2_O as solution B. Then solution B was dropwise added into solution A within 2 min at 180 °C under N_2_ flow. The black solution was kept at 180 °C for another 5 min. After cooling to room temperature, the black solution was washed with ethyl acetate three times and centrifugated. The obtained black powder was stored under vacuum condition. The XRPD pattern of OP-180 was shown in Figure S18. According to the XRF results, the Os/Pt ratio of OP-180 was 15.3/84.7, confirming the incomplete reduction of Os.


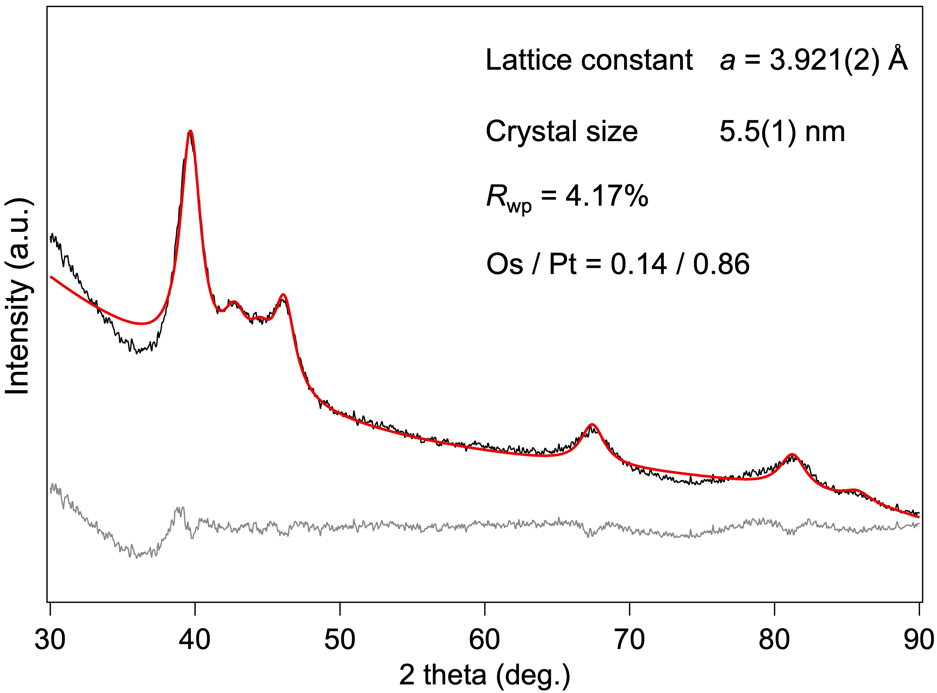


**Figure S18.** XRPD pattern of OP-180 (Os_0.3_Pt_0.7_/C) catalyst (black dots) at 303 K and calculated pattern (red line). The bottom lines show the difference profile (gray). The radiation wavelength was 1.54056 Å.

**3.3 Synthesis of Os_0.3_Pt_0.7_ (OP-NaBH_4_) with strong reducibility.**

To synthesize Os_0.3_Pt_0.7_, the mixture of 86.6 mg K_2_OsCl_6_ and 217.5 mg H_2_PtCl_6_·6H_2_O was dissolved in 100 ml H_2_O. The fresh NaBH_4_ (454 mg) aqueous solution was rapidly added to the suspension at 60 °C. After adding, the NPs solution was kept at 60 °C for 10 min then cooled to room temperature. The suspension was centrifugated and the concentrate was washed with deionized H_2_O several times to remove excess NaBH_4_ and other byproducts. Finally, black powder was collected by vacuum drying. The XRPD pattern of OP-NaBH_4_ was shown in Figure S19. According to the XRD Rietveld refinement results, the Os/Pt ratio of OP- NaBH_4_ was 39.9/60.1, close to the hcp/fcc ratio, verified the independent nucleation process.

**
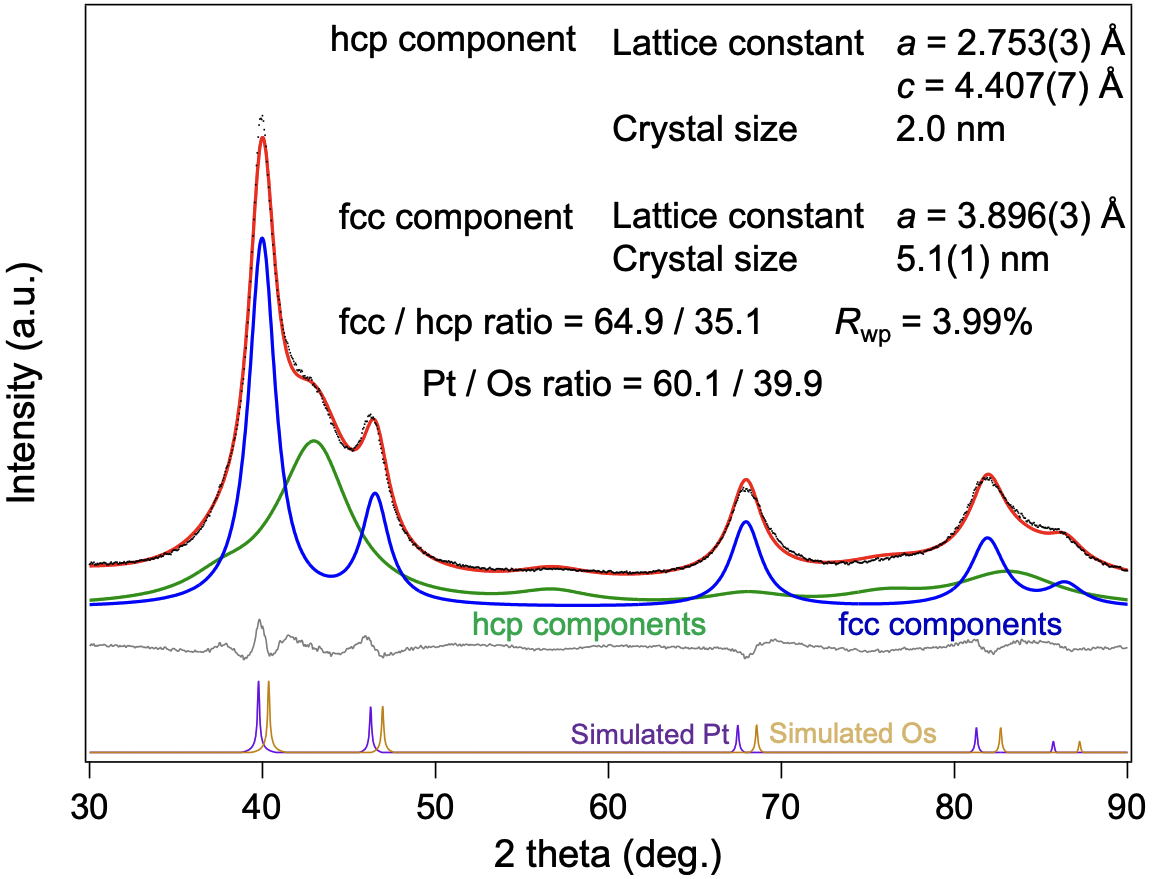
**

**Figure S19.** XRPD pattern of OP-NaBH_4_ catalyst (black dots) at 303 K and calculated pattern (red line). The bottom lines show the difference profile (gray). The radiation wavelength was 1.54056 Å.

**3.4 Synthesis of fcc-Os_0.3_Pt_0.7_/XC-72R.**

The fcc-Os_0.3_Pt_0.7_/XC-72R catalyst was prepared by one-step *in-situ* polyol method. Solution A was 100 ml DEG containing 259 mg XC-72R carbon. 24.1 mg K_2_O_s_Cl_6_ and 25.9 mg H_2_PtCl_6_·6H_2_O were dissolved in 3 ml DEG and 2 ml H_2_O as solution B. Then solution B was dropwise added into solution A within 2 min at 230 °C. The black solution was kept at 230 °C for another 5 min to guarantee the complete reduction. After cooling to room temperature, the black solution was washed with ethyl acetate three times and centrifugated. The obtained black powder was stored under vacuum condition.


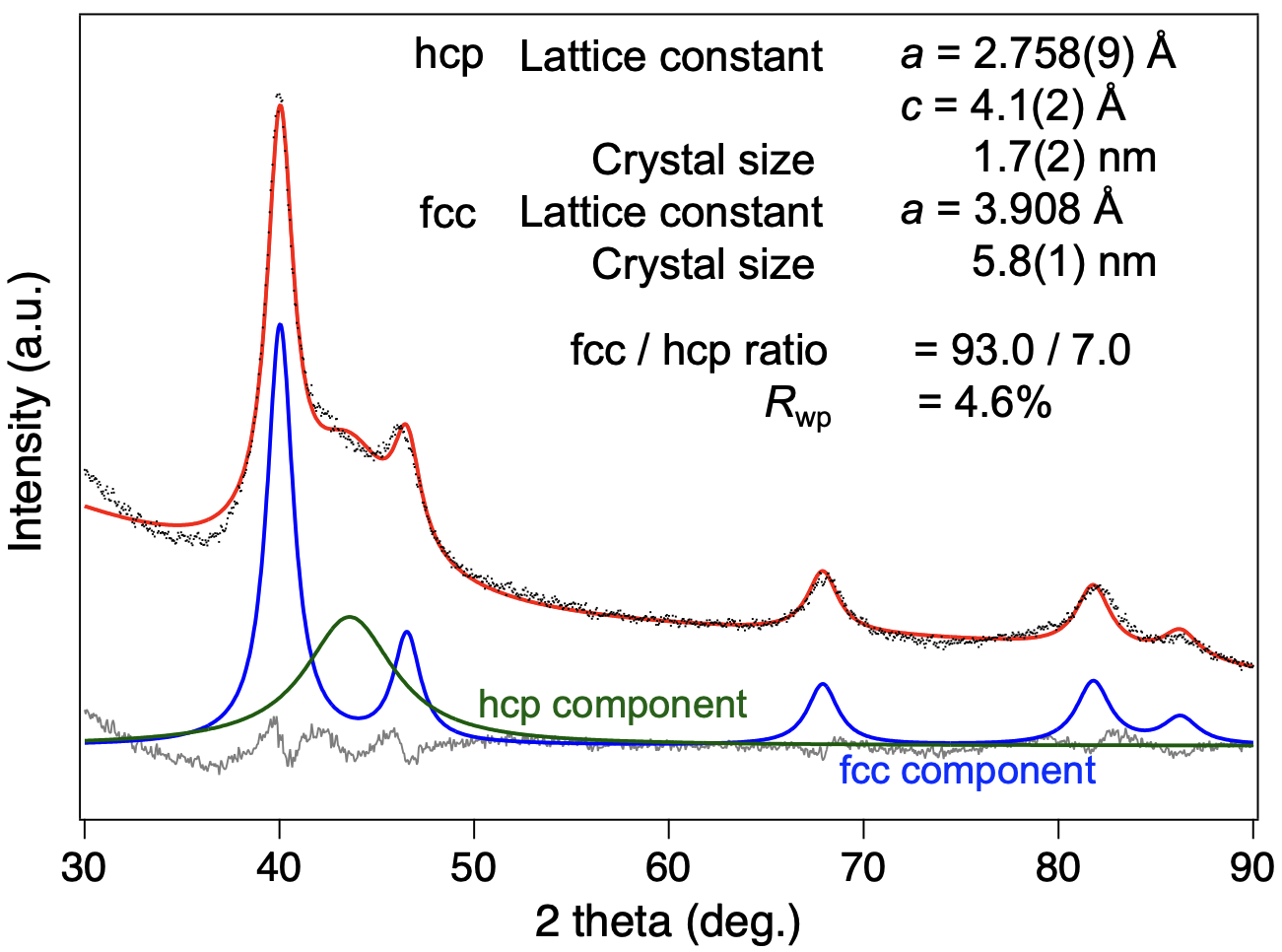


**Figure S20.** XRPD pattern of Os_0.3_Pt_0.7_/XC-72R catalyst (black dots) at 303 K and calculated pattern (red line). The bottom lines show the difference profile (gray). The radiation wavelength was 1.54056 Å.


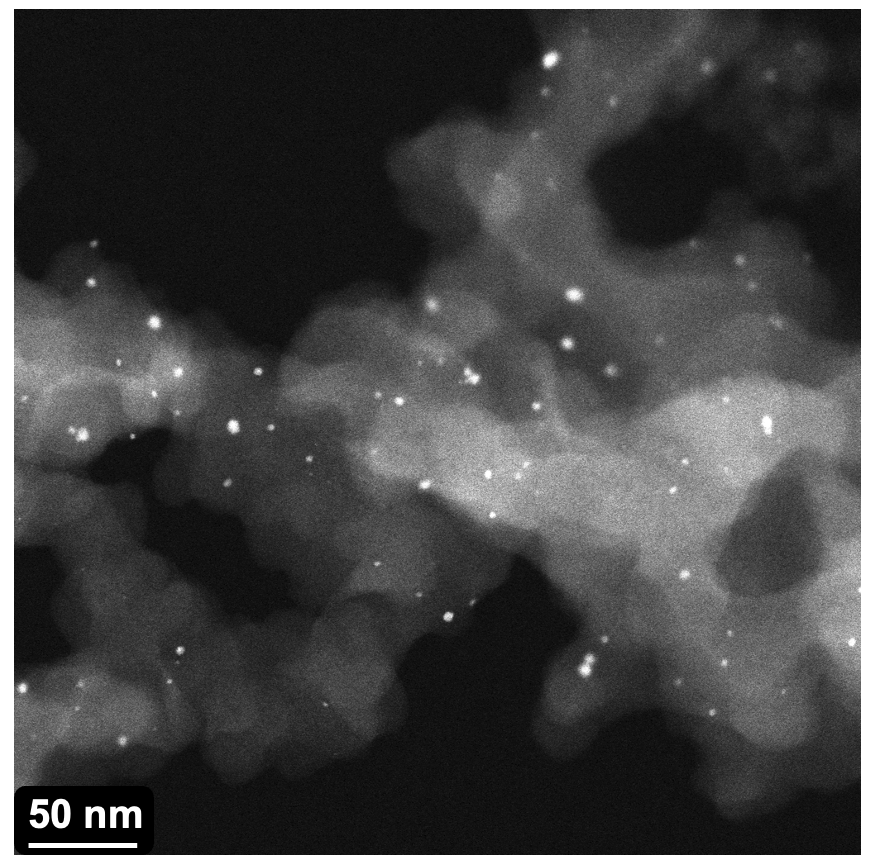


**Figure S21.** HAADF-STEM image of Os_0.3_Pt_0.7_/XC-72R NPs.

**4. HER catalytic performances.**

**Table S5.** The metal loading amounts on carbon determined by XRF measurement and catalyst loading amounts on working electrode.

| Samples | Loading amounts (metal / carbon) | Loading amounts  (metal / electrode) |
| --- | --- | --- |
| Ca. | 10% | 35.4 μg_metal_·cm^-2^ |
| Pt/C | 9.61% | 34.3 μg_metal_·cm^-2^ |
| Os_0.1_Pt_0.9_/C | 10.15% | 36.3 μg_metal_·cm^-2^ |
| Os_0.3_Pt_0.7_/C | 6.87% | 24.5 μg_metal_·cm^-2^ |
| Os_0.5_Pt_0.5_/C | 8.69% | 31.0 μg_metal_·cm^-2^ |
| Os_0.7_Pt_0.3_/C | 8.56% | 30.6 μg_metal_·cm^-2^ |
| Os/C | 7.62% | 27.2 μg_metal_·cm^-2^ |

**Table S6.** Comparison for loading amounts of catalyst on electrode, *η*_10_, and mass activities with other reported Pt-based catalysts.

| Materials | Electrolyte | Mass loading | *η*_10_  (mV) | mass activities  at 20 mV | Reference |
| --- | --- | --- | --- | --- | --- |
| Os_x_Pt_1-x_/C | 0.5 M H_2_SO_4_ | Ca. 2.5 μg_metal_ | 1.0 | 4.2 A·mg_metal_^-1^ | This work |
| PtCuMnP/N-rGo | 0.3 M H_2_SO_4_ | 25 μg | 6.5 | 1.87*10^-3^ A·mg_Pt_^-1^ | [4] |
| PtNi_NPs_/Au_SA_ -NDC | 0.5 M H_2_SO_4_ | 50 μg | 19.1 | 2.0*10^-3^ A·mg_Pt_^-1^ | [5] |
| Pt@PtIr | 0.5 M H_2_SO_4_ | 16 μg | 22 | 0.7 A·mg_Ir+Pt_^-1^ | [6] |
| Pt/PtO_2_/TiO_2_ | 0.5 M H_2_SO_4_ | Ca. 100 μg | 12 | 0.1 A·mg_Pt_^-1^ | [7] |
| PtW/C | 0.5 M H_2_SO_4_ | 4 μg_Pt_ | 19.4 | 0.57 A·mg_Pt_^-1^ | [8] |
| Fct-FePt | 0.5 M H_2_SO_4_ | 45.5 μg_Pt_ | 15.8 | 0.31 A·mg_Pt_^-1^ | [9] |
| PtRu@RFCS | 0.5 M H_2_SO_4_ | 25 μg | 19.7 | 28.6*10^-3^ A·mg_Pt_^-1^ | [10] |
| Pd@PtCu/C | 0.5 M H_2_SO_4_ | Ca. 7.3 μg_Pt_ | 19 | 1.7 A·mg_Pt_^-1^ | [11] |

**Table S7.** Particle size and percent surface atoms for Os_x_Pt_1-x_/Al_2_O_3_.^[12]^

| Samples | Particle size (nm) | Percent surface atoms (%) |
| --- | --- | --- |
| Pt/C | 5.1 | 15.41 |
| Os_0.1_Pt_0.9_/C | 5.3 | 14.86 |
| Os_0.3_Pt_0.7_/C | 3.2 | 23.42 |
| Os_0.5_Pt_0.5_/C | 2.6 | 28.79 |
| Os_0.7_Pt_0.3_/C | 3.7 | 20.87 |
| Os/C | 1.5 | 44.84 |


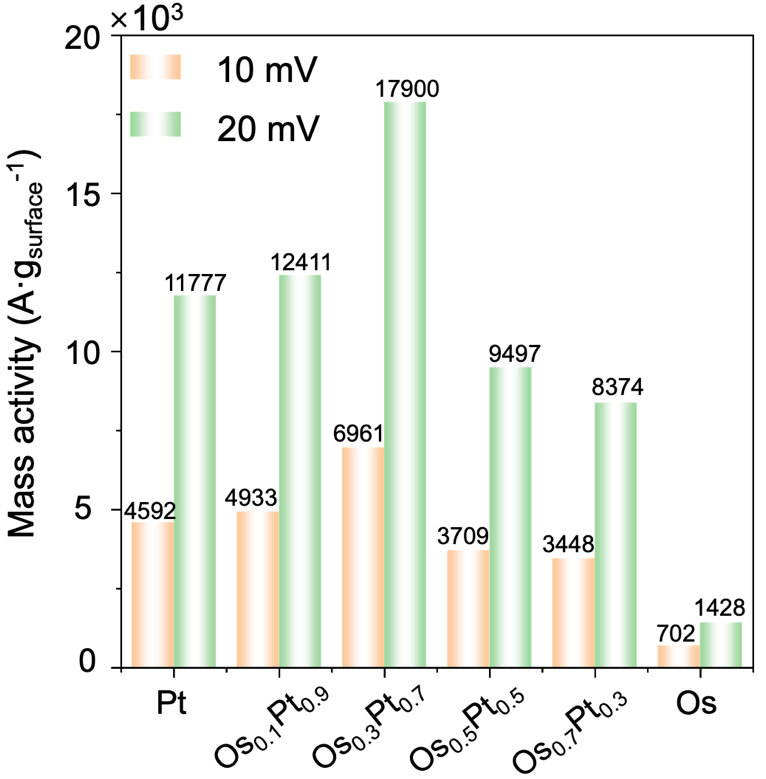


**Figure S22.** Surface mass activities at 10 and 20 mV overpotentials Polarization curves of Os_x_Pt_1-x_/C in 0.5 M H_2_SO_4_.

**Table S8.** Comparison for surface mass activities of Os_0.3_Pt_0.7_/C with other Pt-based single atoms catalysts.

| Materials | Electrolyte | Pt wt% | Surface mass activities at 20 mV  (A·mg_Pt_^-1^) | Reference |
| --- | --- | --- | --- | --- |
| Os_0.3_Pt_0.7_/C | 0.5 M H_2_SO_4_ | 1.6 (surface atoms) | 17.9 | This work |
| NGA-COF@Pt | 0.5 M H_2_SO_4_ | 4.32 | 2.3 | [13] |
| Pt_SA_/α-MoC_1−_*_x_*@C | 0.5 M H_2_SO_4_ | 0.75 | 5.3 | [14] |
| Pt@Mn-SAs/N-C | 0.5 M H_2_SO_4_ | 1.98 | 0.18 | [15] |
| Pt_1_/NMHCS | 0.5 M H_2_SO_4_ | 2.1 | 0.2 | [16] |
| Pt@VNC | 0.5 M H_2_SO_4_ | 3.38 | 5.7 | [17] |
| Pt-MoAl_1-x_B | 0.5 M H_2_SO_4_ | 0.9 | 0.5 | [18] |
| Pt-SAs/WS_2_ | 0.5 M H_2_SO_4_ | 4.1 | 11.9 | [19] |
| Ti_3_C_2_T*_x_*-Pt_SA_ | 0.5 M H_2_SO_4_ | 0.84 | 2.0 | [20] |


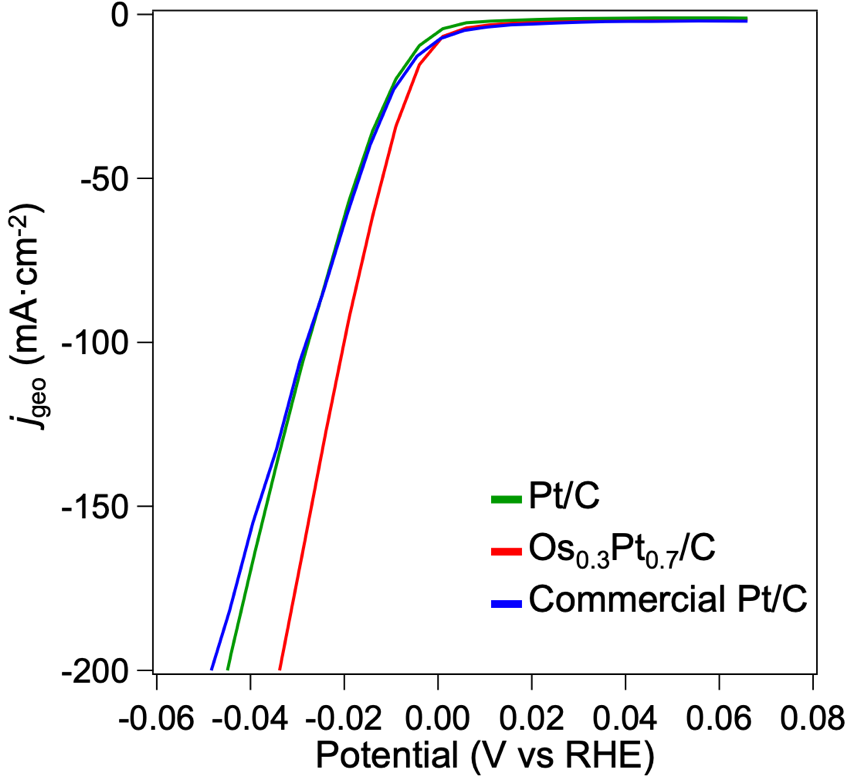


**Figure S23.** Polarization curves of Os_0.3_Pt_0.7_/C, Pt/C by polyol method and commercial Pt/C in 0.5 M H_2_SO_4_.

**
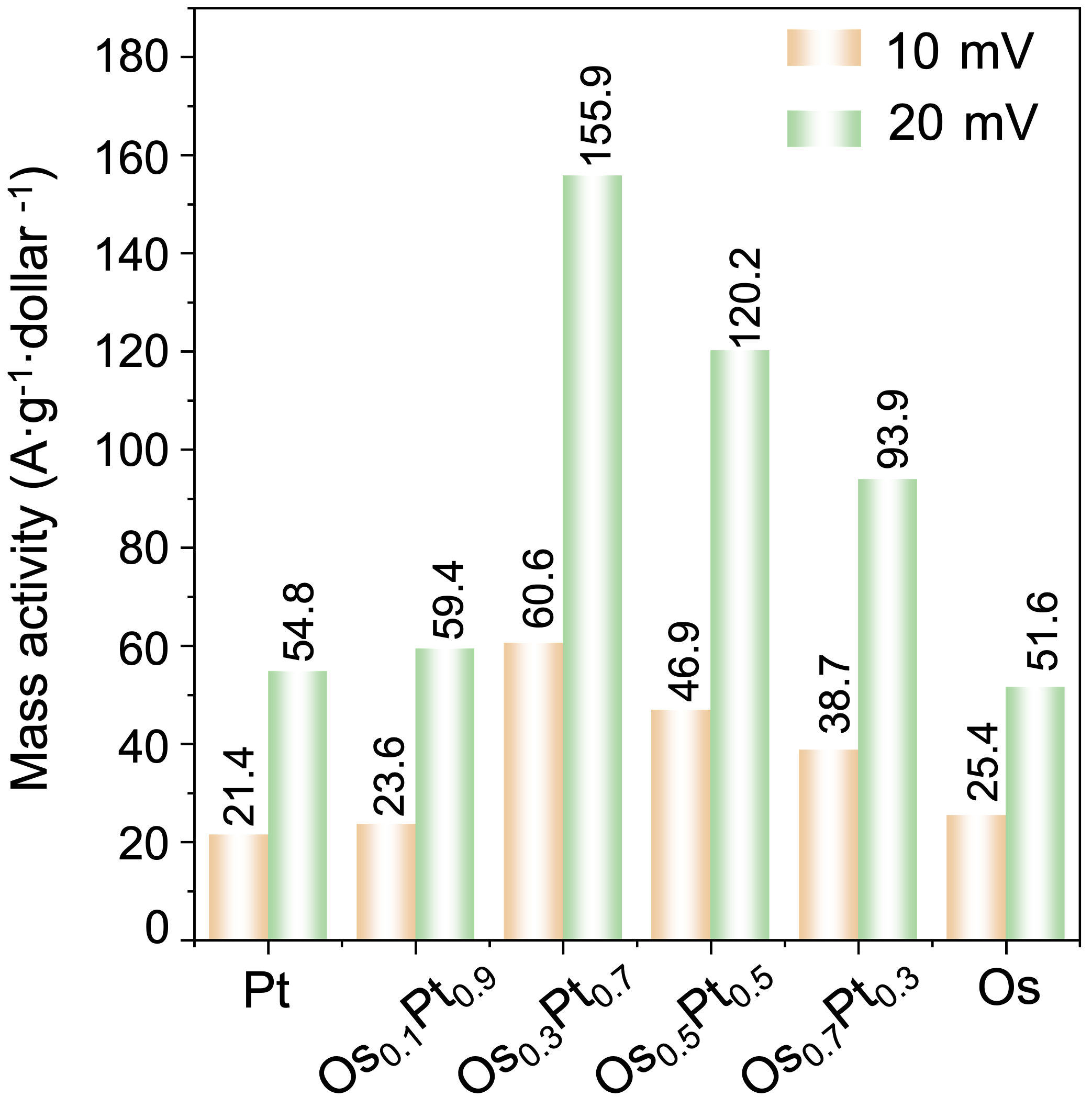
**

Figure S24. Cost-performance for Os_x_Pt_1-x_/C in 0.5 M H_2_SO_4_ evaluated by mass activities at overpotentials of 10 mV and 20 mV.


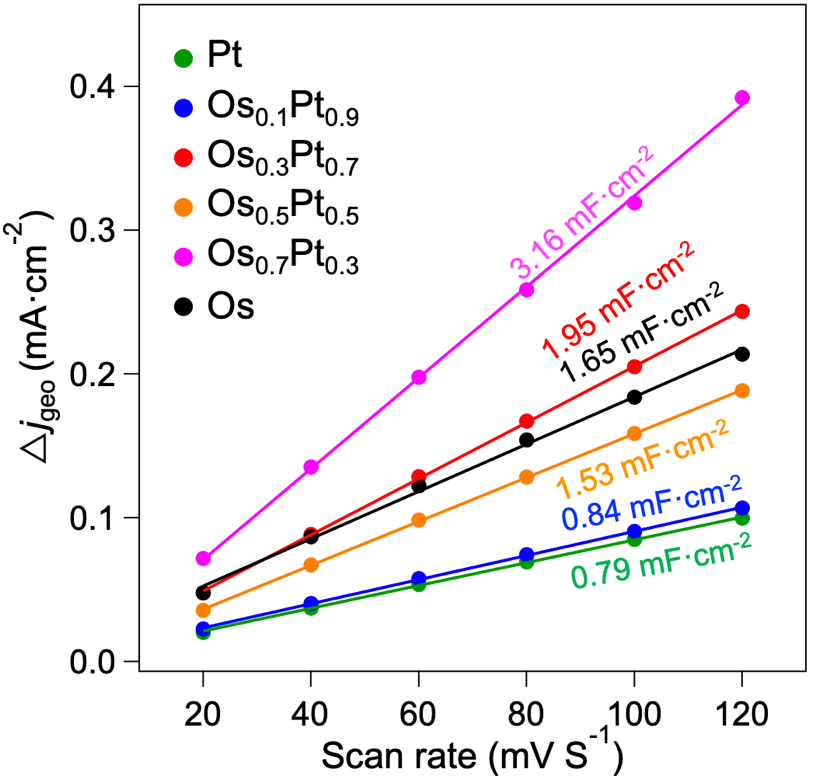


**Figure S25.** Capacitive current plot under different scan rates of Os_x_Pt_1-x_/C in 0.5 M H_2_SO_4_.


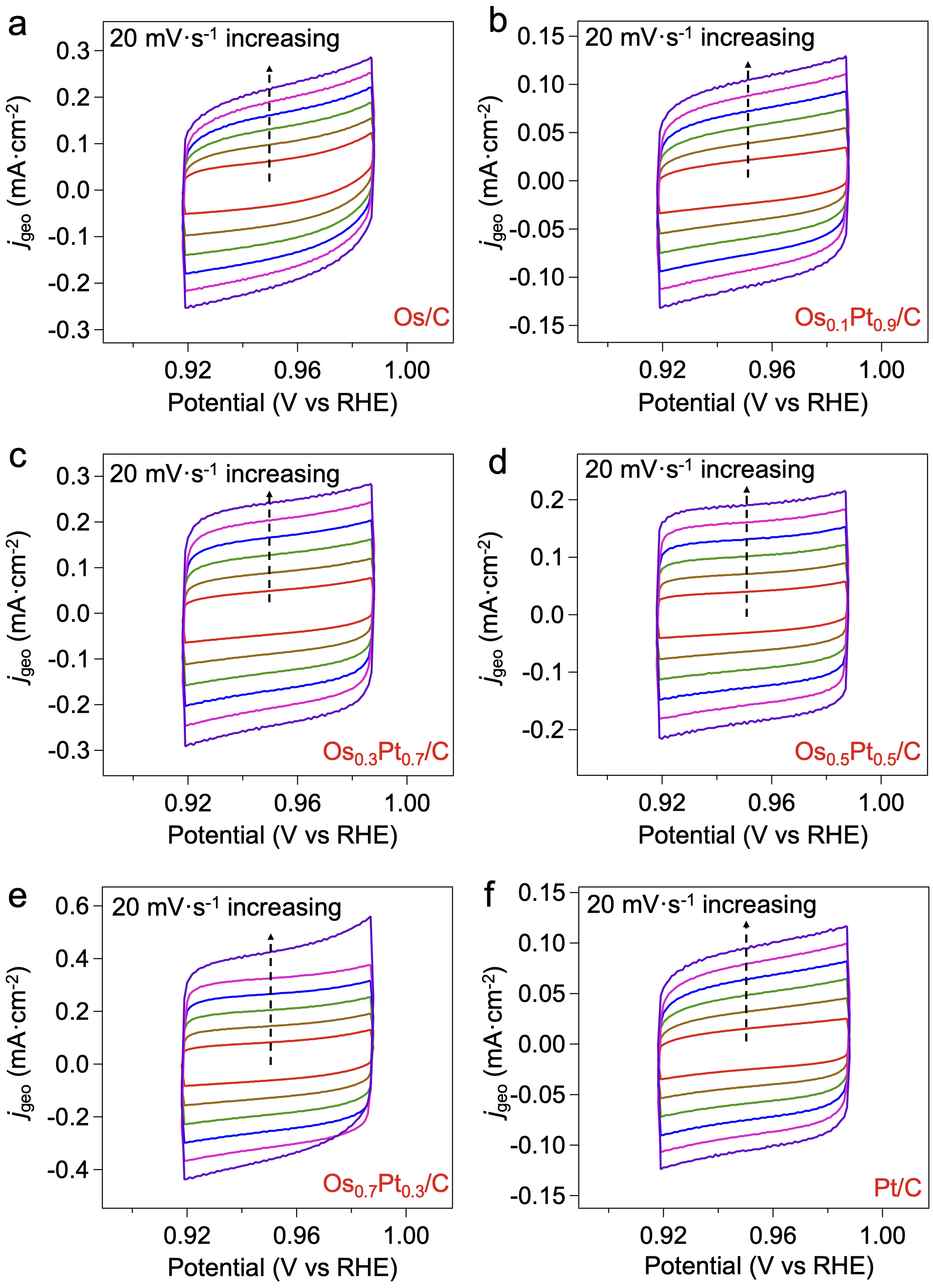


**Figure S26.** CV curves of Os_x_Pt_1-x_/C in 0.5 M H_2_SO_4_ at the scan rates of 20, 40, 60, 80, 100 and 120 mV⋅s^-1^. **a)** Os/C, **b)** Os_0.1_Pt_0.9_/C, **c)** Os_0.3_Pt_0.7_/C, **d)** Os_0.5_Pt_0.5_/C, **e)** Os_0.7_Pt_0.3_/C **f)** Pt/C.

**
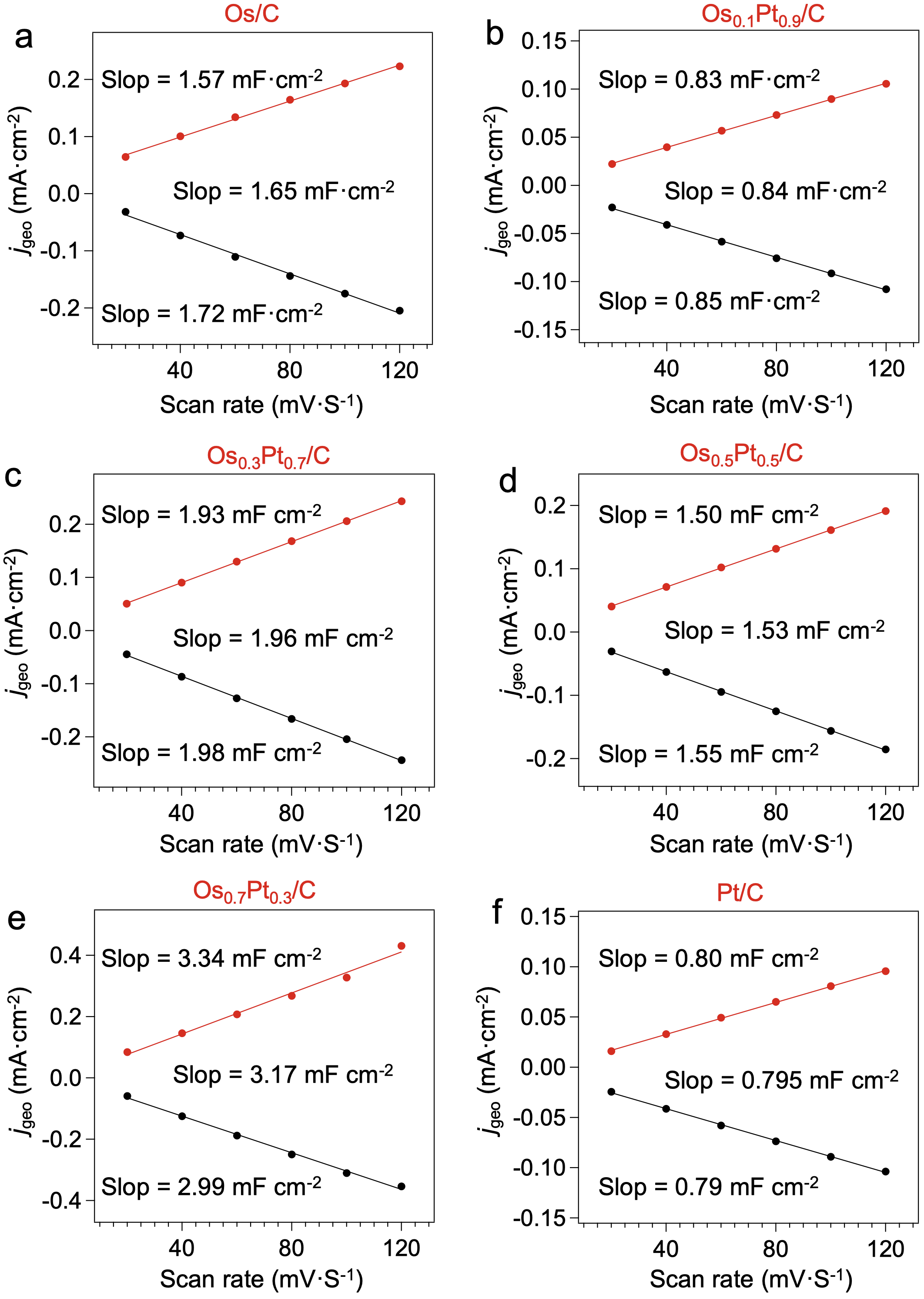
**

**Figure S27.** The current densities measured at 0.235 V with different scan rates of Os_x_Pt_1-x_/C in 0.5 M H_2_SO_4_. **a)** Os/C, **b)** Os_0.1_Pt_0.9_/C, **c)** Os_0.3_Pt_0.7_/C, **d)** Os_0.5_Pt_0.5_/C, **e)** Os_0.7_Pt_0.3_/C **f)** Pt/C.


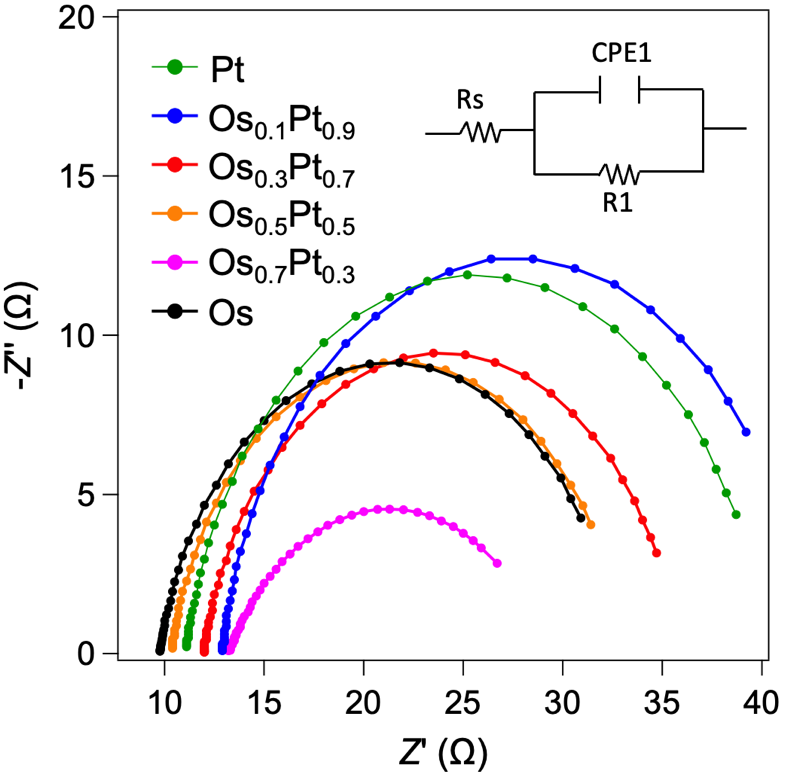


**Figure S28.** Electrochemical impedance spectroscopy (EIS) plots and corresponding equivalent circuits of Os_x_Pt_1-x_/C in 0.5 M H_2_SO_4_.


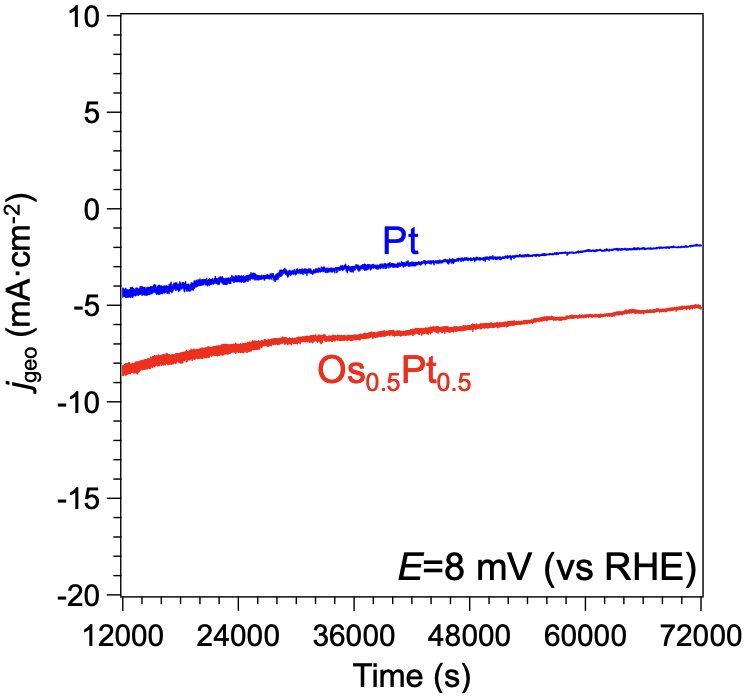


**Figure S29.** Stability tests of fcc-Os_0.5_Pt_0.5_ and pure Pt NPs in 0.5 M H_2_SO_4_.


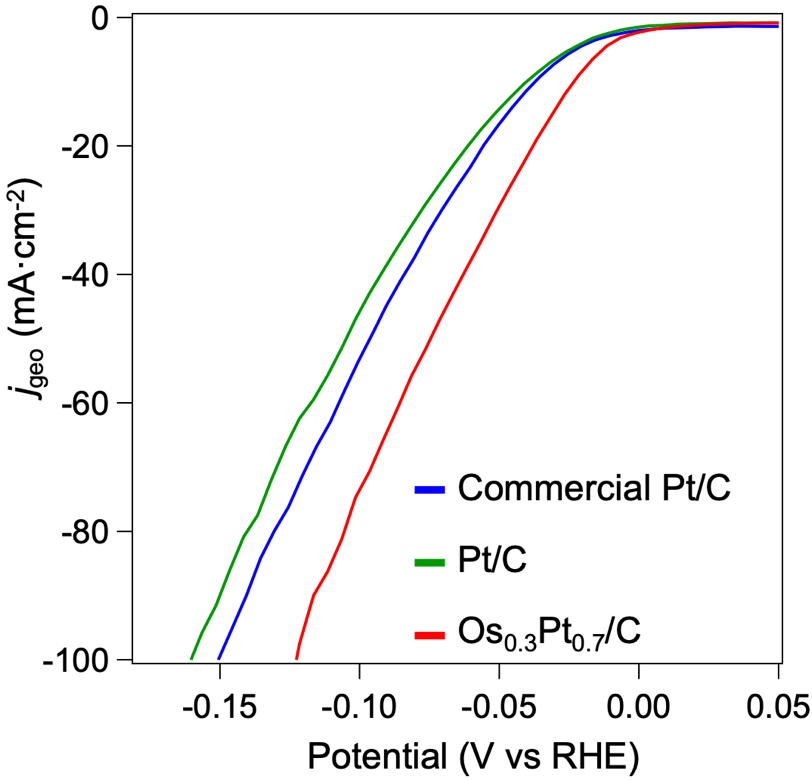


**Figure S30.** Polarization curves of Os_0.3_Pt_0.7_/C, Pt/C by polyol method and commercial Pt/C in 1 M KOH.


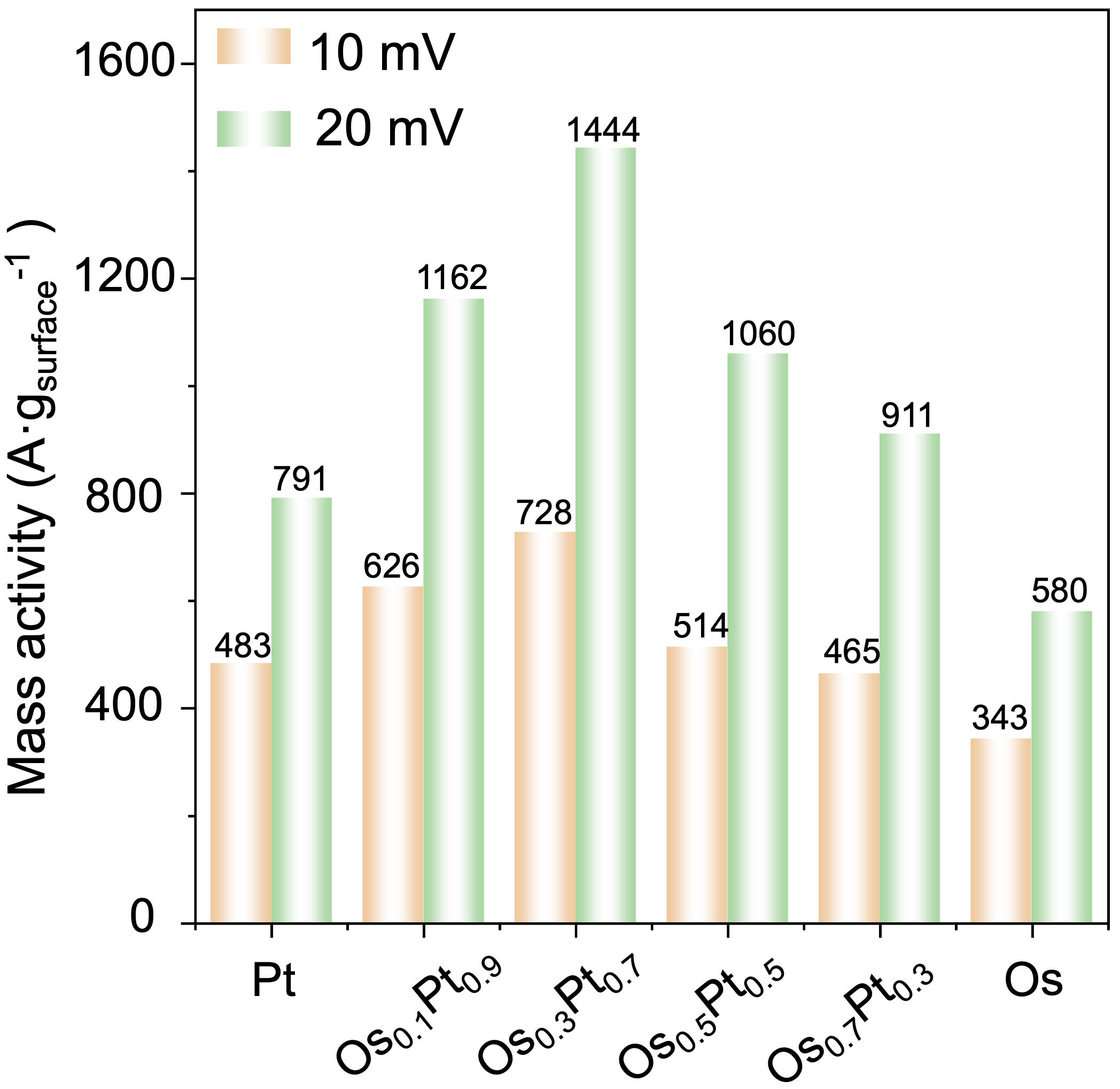


**Figure S31.** Surface mass activities at 10 and 20 mV overpotentials Polarization curves of Os_x_Pt_1-x_/C in 1 M KOH.


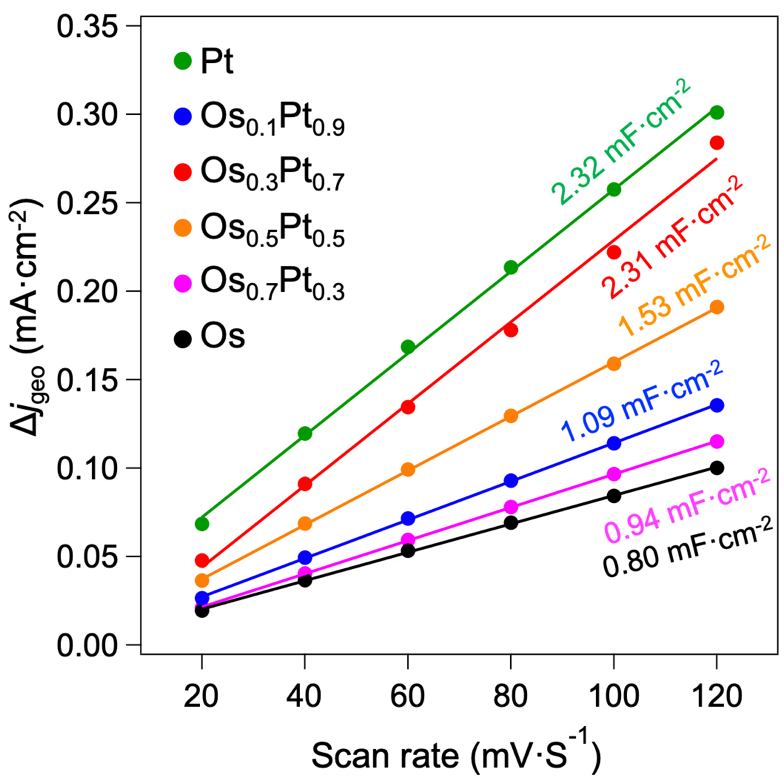


**Figure S32.** Capacitive current plot under different scan rates of Os_x_Pt_1-x_/C in 1 M KOH.


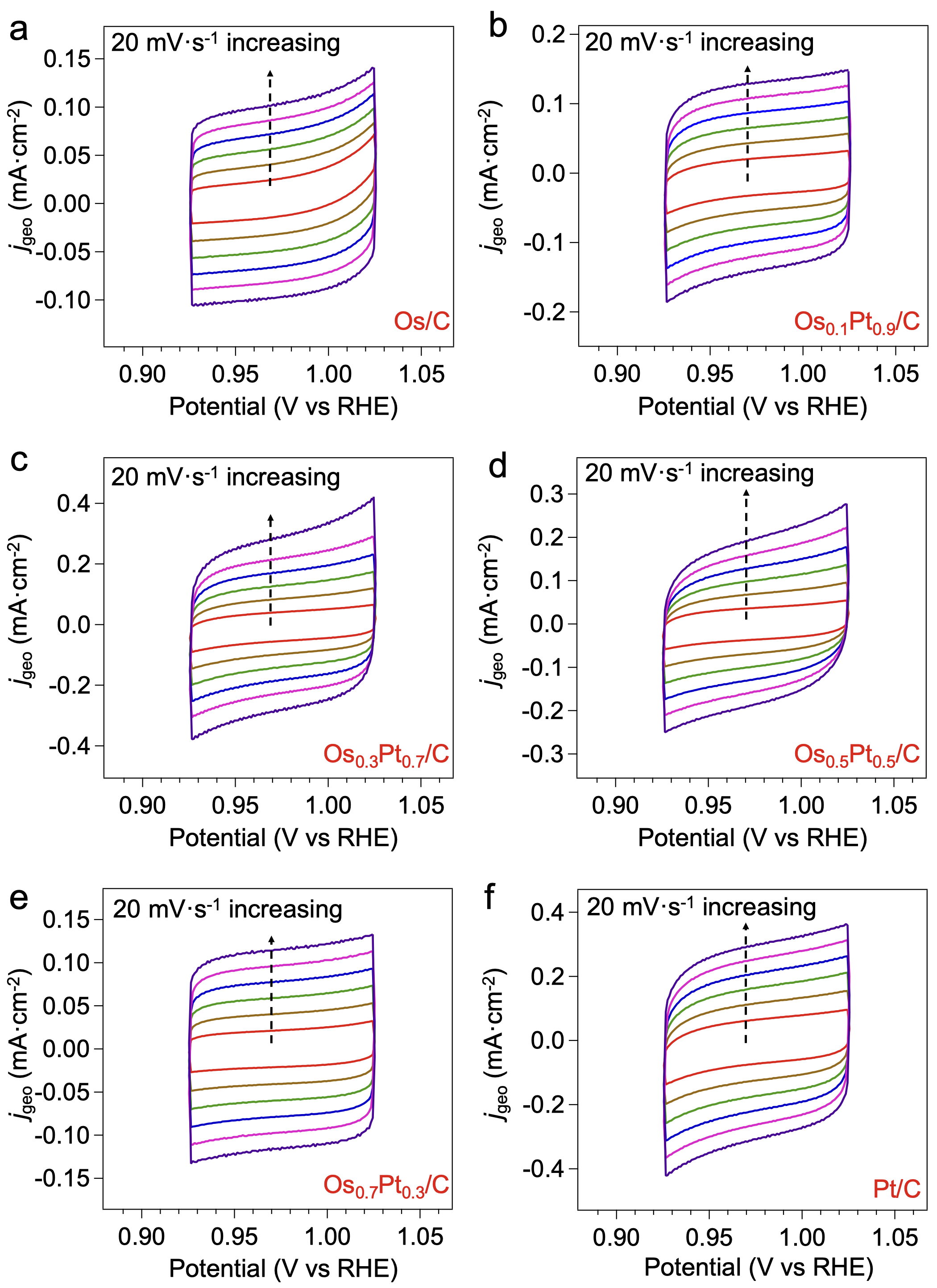


**Figure S33.** CV curves of Os_x_Pt_1-x_/C in 1 M KOH at the scan rates of 20, 40, 60, 80, 100 and 120 mV⋅s^-1^. **a)** Os/C, **b)** Os_0.1_Pt_0.9_/C, **c)** Os_0.3_Pt_0.7_/C, **d)** Os_0.5_Pt_0.5_/C, **e)** Os_0.7_Pt_0.3_/C **f)** Pt/C.


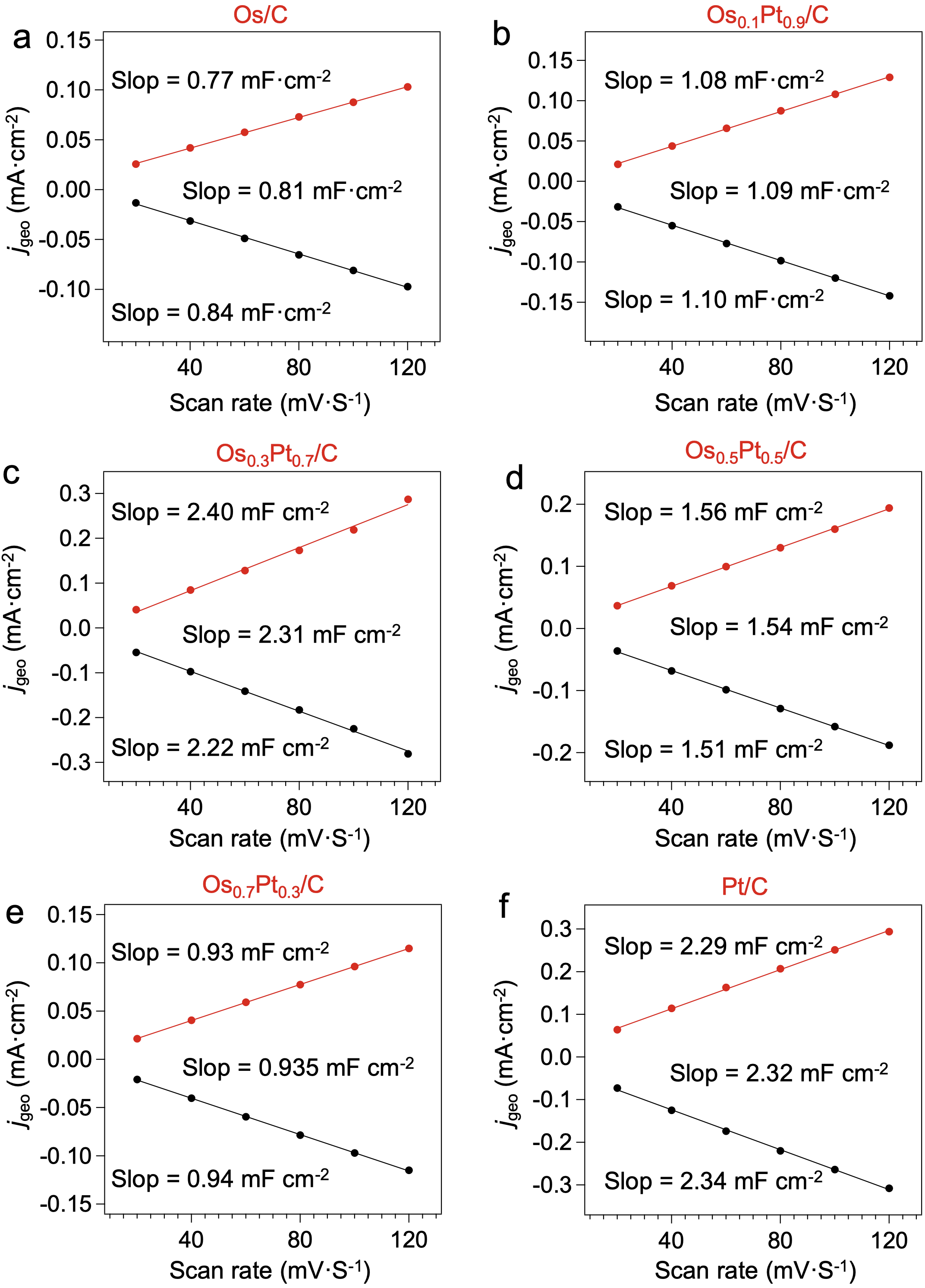


**Figure S34.** The current densities measured at 0.235 V with different scan rates of Os_x_Pt_1-x_/C in 1 M KOH. **a)** Os/C, **b)** Os_0.1_Pt_0.9_/C, **c)** Os_0.3_Pt_0.7_/C, **d)** Os_0.5_Pt_0.5_/C, **e)** Os_0.7_Pt_0.3_/C **f)** Pt/C.


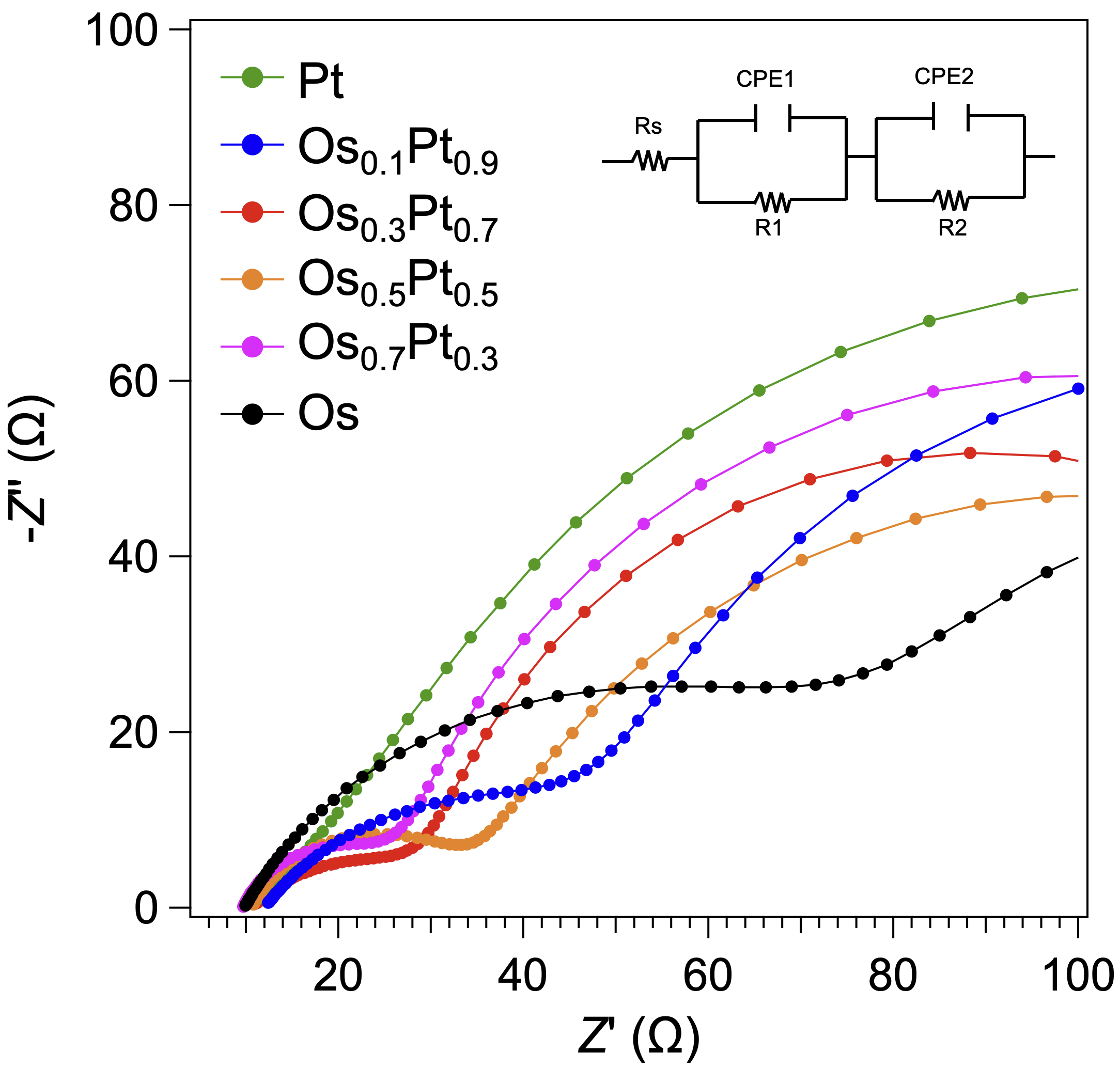


**Figure S35.** Electrochemical impedance spectroscopy (EIS) plots and corresponding equivalent circuits of Os_x_Pt_1-x_/C in 1 M KOH.


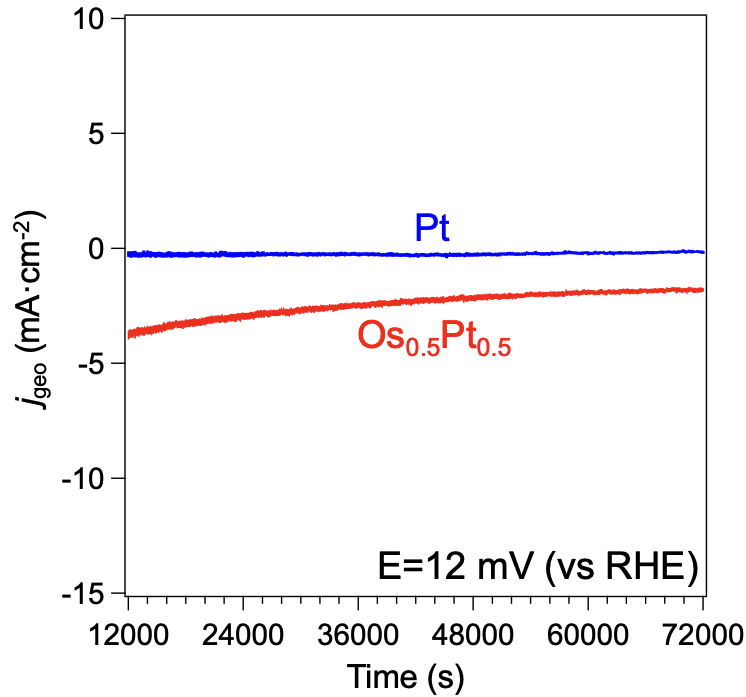


**Figure S36.** Stability tests of fcc-Os_0.5_Pt_0.5_ and pure Pt NPs in 1.0 M KOH.

**5. Fitting data of XPS spectra of Os, Pt and Os_0.5_Pt_0.5_ NPs.**

**Table S9.** Binding energy of specific peaks of Os/C, Pt/C, Os_0.5_Pt_0.5_/C and Os_0.3_Pt_0.7_/C.

| Nanoparticles | Os 4f 5/2 | Os 4f 7/2 | Pt 4f 5/2 | Pt 4f 7/2 |
| --- | --- | --- | --- | --- |
| Os/C | 54.2 | 51.4 | - | - |
| Pt/C | - | - | 74.5 | 71.1 |
| Os_0.5_Pt_0.5_/C | 53.6 | 50.9 | 74.9 | 71.6 |
| Os_0.3_Pt_0.7_/C | 53.5 | 50.8 | 74.6 | 71.2 |

**6. DFT calculations.**


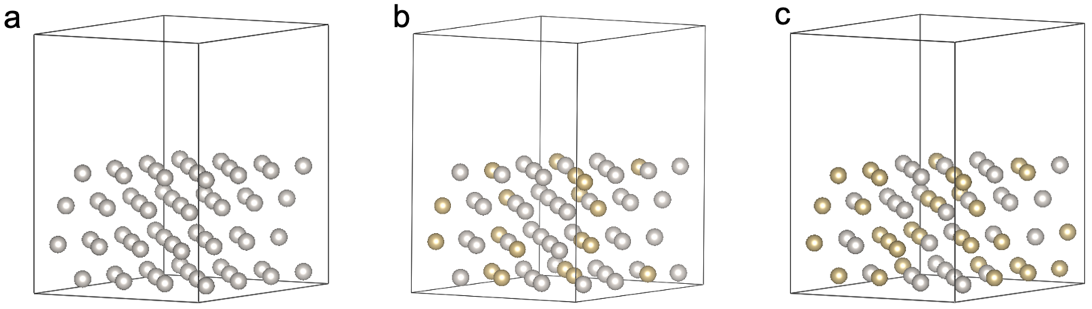


**Figure S37. a)** Surface-DOS computational model of Pt_32_ for pure Pt (111). **b)** Surface-DOS computational model of Os_16_Pt_16_ for Os_0.3_Pt_0.7_ (111). **c)** Surface-DOS computational model of Os_16_Pt_16_ for Os_0.5_Pt_0.5_ (111).


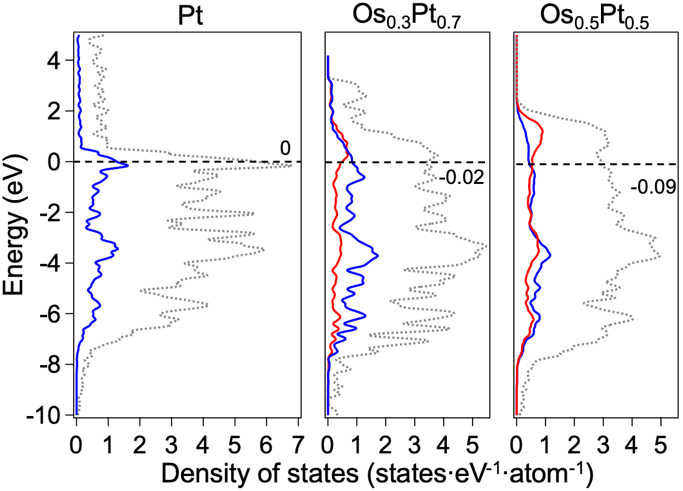


**Figure S38**. DOS of the top layer for Pt (111), fcc-Os_0.3_Pt_0.7_ (111) and fcc-Os_0.5_Pt_0.5_ (111) NPs in total range. The grey curves show the total DOS of each sample, and the red and blue curves show the d_z_^2^ partial DOS of Os and Pt elements, respectively. The black dashed line shows the Fermi level of each sample.


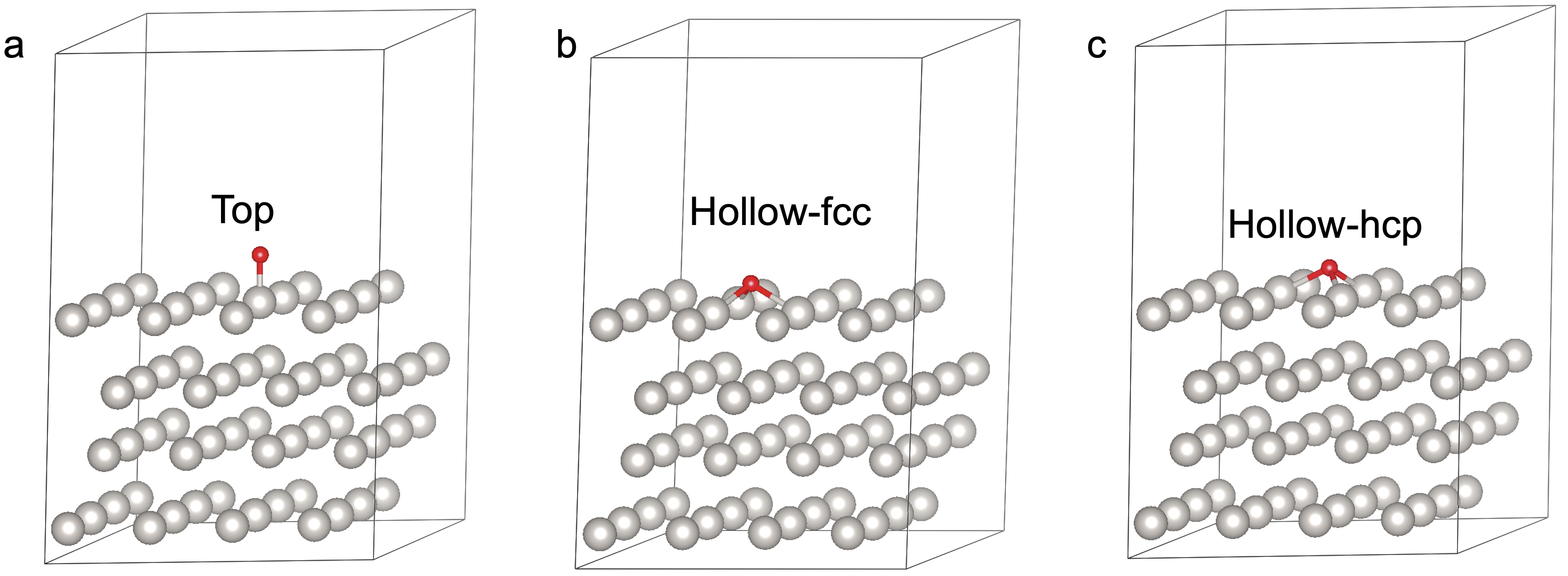


**Figure S39**. H adsorption energy calculation models at different sites for pure Pt (111).


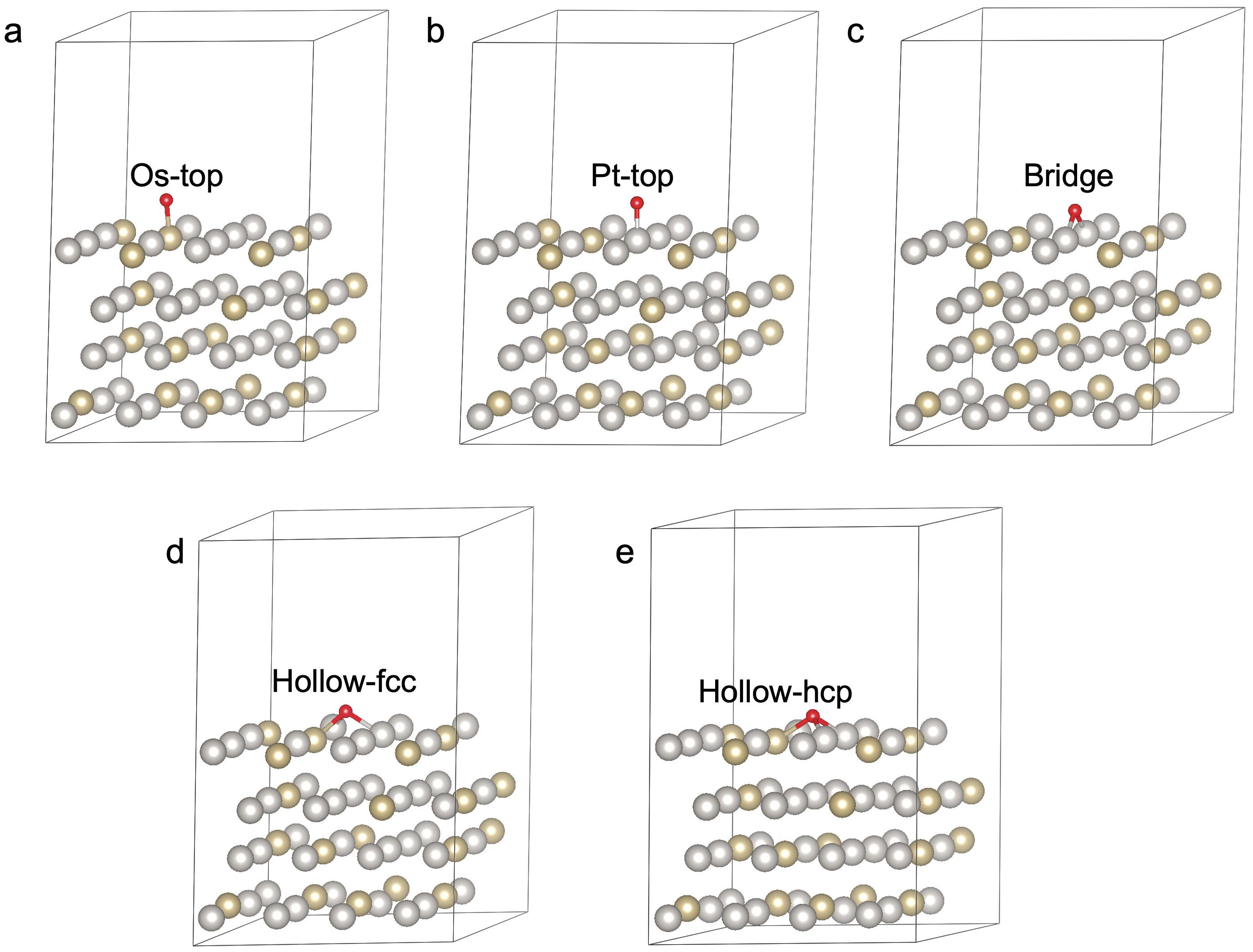


**Figure 40.** H adsorption energy calculation models at different sites for Os_0.3_Pt_0.7_ (111).


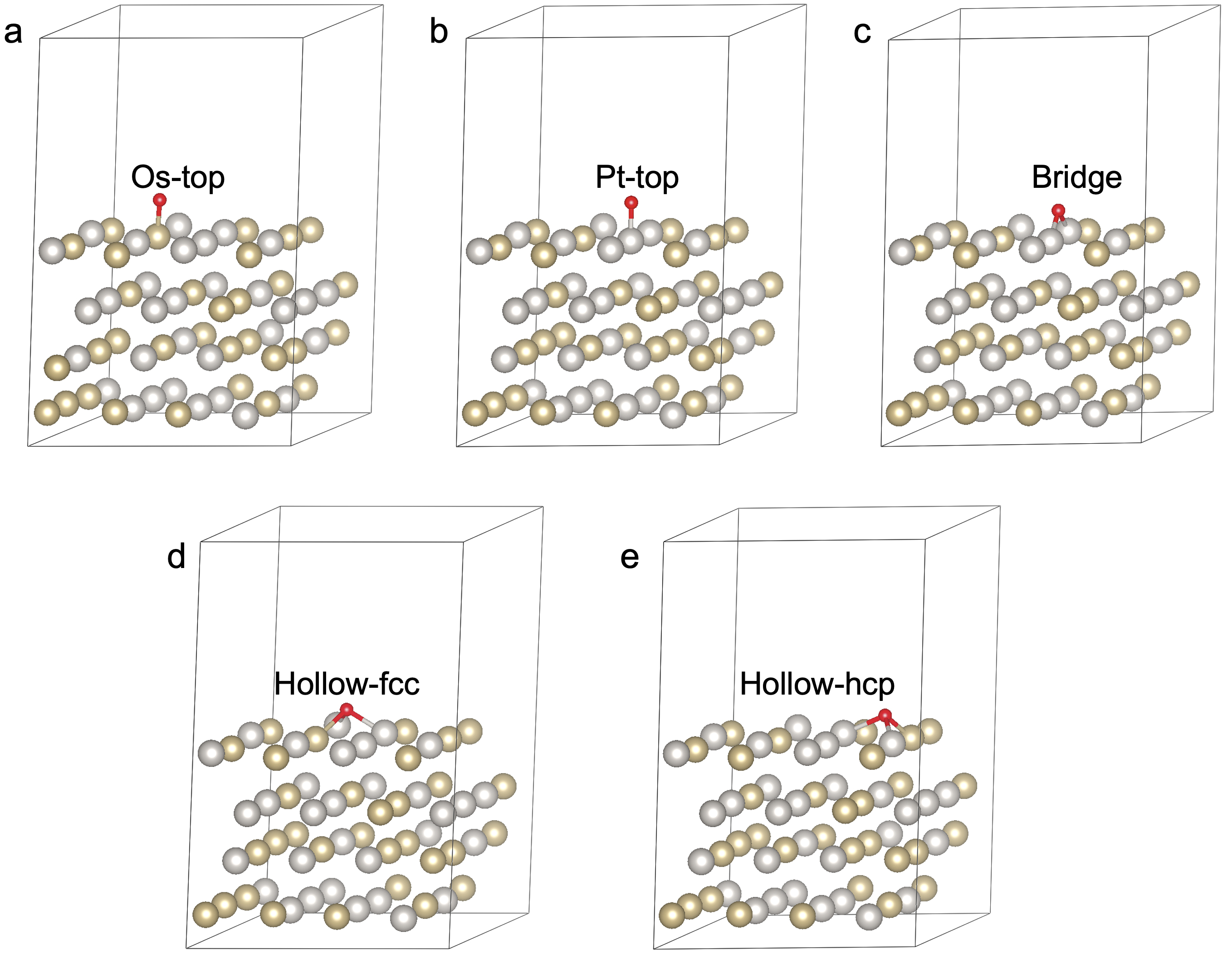


**Figure S41**. H adsorption energy calculation models at different sites for Os_0.5_Pt_0.5_ (111).

**Table S10.** The calculated H adsorption energy at different sites for Pt (111), fcc-Os_0.3_Pt_0.7_ (111) and fcc-Os_0.5_Pt_0.5_ (111) NPs.

| Pt | Sites | top | hollow |  |  |
| --- | --- | --- | --- | --- | --- |
|  | Energy (eV) | -0.2615 | -0.2225 |  |  |
| fcc-Os_0.3_Pt_0.7_ | Sites | Os-top | Pt-top | bridge | hollow |
|  | Energy (eV) | -0.3075 | -0.1515 | -0.1685 | -0.1495 |
| fcc-Os_0.5_Pt_0.5_ | Sites | Os-top | Pt-top | bridge | hollow |
|  | Energy (eV) | -0.2665 | -0.0975 | -0.0825 | -0.2135 |

**Table S11.** The calculated average *ΔG*_H*_ of the couple sites for Pt (111), fcc-Os_0.3_Pt_0.7_ (111) and fcc-Os_0.5_Pt_0.5_ (111) NPs.

| Pt | Couple sites | top-top | hollow-hollow |
| --- | --- | --- | --- |
|  | Energy (eV) | -0.2615 | -0.2225 |
| fcc-Os_0.3_Pt_0.7_ | Couple sites | top-top | hollow-hollow |
|  | Energy (eV) | -0.1515 | -0.1495 |
| fcc-Os_0.5_Pt_0.5_ | Couple sites | top-top | bridge-top |
|  | Energy (eV) | -0.0975 | -0.09 |

**Reference**

[1] Kresse, G., Furthmuller, J., Efficient iterative schemes for ab initio total-energy calculations using a plane-wave basis set, **1996**, Phys. Rev. B, 54, 11169-11186, <https://doi.org/10.1103/PhysRevB.54.11169>.

[2] Perdew, J. P., Burke, K., Ernzerhof, M., Generalized Gradient Approximation Made Simple, **1996**, *Phys. Rev. Lett.*, 77, 3865-3868, <https://doi.org/10.1103/PhysRevLett.77.3865>.

[3] Sidorov, E., Kutyrev, A., Chubarov, V., Zhitova, E., Platinum mineralization of the Epilchik Ural-Alaskan type zoned complex (Far East Russia), **2020**, *Miner. Deposita*, 56, 143-160, https://doi.org/10.1007/s00126-020-01008-1.

[4] Basumatary, P., Choi, J. H., Konwar, D., Ramchiary, A., Han, B., Yoon, Y. S., Hierarchical PtCuMnP Nanoalloy for Efficient Hydrogen Evolution and Methanol Oxidation, **2024**, *Small Methods*, 8, e2301651, <https://doi.org/10.1002/smtd.202301651>.

[5] Le, T. D., Kim, D. S., Tran, T. V., Urupalli, B., Shin, G. S., Oh, G. J., Yu, Y. T., Electronic Structure Engineering of Pt–Ni Alloy NPs by Coupling of Gold Single Atoms on N-Doped Carbon for Highly Efficient Oxygen Reduction Reaction and Hydrogen Evolution Reaction, **2024**, *Small*, 20, e2311971, <https://doi.org/10.1002/smll.202311971>.

[6] Liu, C., Wei, Z., Cao, M., Cao, R., Pt nanoparticles with a PtIr alloy surface structure exhibit excellent stability toward acidic hydrogen evolution reaction. **2024**, *Nano Res.*, 17, 4844-4849, https://doi.org/10.1007/s12274-024-6454-3.

[7] Tang, X., Yu, A., Yang, Q. *et al.* Significance of Epitaxial Growth of PtO2 on Rutile TiO2 for Pt/TiO2 Catalysts, **2024**, *J. Am. Chem. Soc.*, 146, 3764-3772, <https://doi.org/10.1021/jacs.3c10659>.

[8] Kobayashi, D., Kobayashi, H., Wu, D. *et al.* Significant Enhancement of Hydrogen Evolution Reaction Activity by Negatively Charged Pt through Light Doping of W, **2020**, *J. Am. Chem. Soc.*, 142, 17250-17254, <https://doi.org/10.1021/jacs.0c07143>.

[9] Li, Q., Wu, L., Wu, G. *et al.* New Approach to Fully Ordered fct-FePt Nanoparticles for Much Enhanced Electrocatalysis in Acid, **2015**, *Nano Lett.*, 15, 2468-2473, <https://doi.org/10.1021/acs.nanolett.5b00320>.

[10] Li, K., Li, Y., Wang, Y., Ge, J., Liu, C., Xing, W., Enhanced electrocatalytic performance for the hydrogen evolution reaction through surface enrichment of platinum nanoclusters alloying with ruthenium in situ embedded in carbon, **2018**, *Energy & Environ. Sci.*, 11, 1232-1239, <https://doi.org/10.1039/C8EE00402A>.

[11] Bao, M., Amiinu, I. S., Peng, T. *et al.* Surface Evolution of PtCu-Alloy-Shell Over Pd-Nanocrystals Leads to Superior Hydrogen Evolution and Oxygen Reduction Reactions, **2018**, *ACS* *Energy Lett*., 3, 940-945, <https://doi.org/10.1021/acsenergylett.8b00330>.

[12] Bond, G., Metal Catalysed Reactions of Hydrocarbons, Springer, **2005**, page 36.

[13] Zhang, Z., Zhang, Z., Chen, C. *et al.* Single-atom platinum with asymmetric coordination environment on fully conjugated covalent organic framework for efficient electrocatalysis, **2024**, *Nat. Commun.*, 15, 2556, https://doi.org/10.1038/s41467-024-46872-x.

[14] Wang, W., Wu, Y., Lin, Y. *et al.* Confining Zero-Valent Platinum Single Atoms in α-MoC1−x for pH-Universal Hydrogen Evolution Reaction, **2021**, *Adv. Funct. Mater.*, 32, 2108464, <https://doi.org/10.1002/adfm.202108464>.

[15] Gong, L., Zhu, J., Xia, F. *et al.* Marriage of Ultralow Platinum and Single-Atom MnN4 Moiety for Augmented ORR and HER Catalysis, **2023**, *ACS Catal.*, 13, 4012-4020, <https://doi.org/10.1021/acscatal.2c06340>.

[16] Kuang, P., Wang, Y., Zhu, B. *et al.* Pt Single Atoms Supported on N-Doped Mesoporous Hollow Carbon Spheres with Enhanced Electrocatalytic H2-Evolution Activity, **2021**, *Adv. Mater.*, 33, e2008599, <https://doi.org/10.1002/adma.202008599>.

[17] Jin, H., Ha, M., Kim, M. G., Lee, J. H., Kim, K. S., Engineering Pt Coordination Environment with Atomically Dispersed Transition Metal Sites Toward Superior Hydrogen Evolution, **2023**, *Adv. Energy Mater.*, 13, 2204213, <https://doi.org/10.1002/aenm.202204213>.

[18] Park, S. J., Nguyen, T. H., Tran, D. T., Dinh, V. A., Lee, J. H., Kim, N. H., Delaminated MBene sheets beyond usual 2D transition metal materials for securing Pt single atoms to boost hydrogen evolution, **2023**, *Energy & Environ. Sci.*, 16, 4093-4104, <https://doi.org/10.1039/D3EE01314F>.

[19] Shi, Y., Ma, Z. R., Xiao, Y. Y. *et al.* Electronic metal–support interaction modulates single-atom platinum catalysis for hydrogen evolution reaction, **2021**, *Nat. Commun*., 12, 3021, https://doi.org/10.1038/s41467-021-23306-6.

[20] Zhang, J., Wang, E., Cui, S., Yang, S., Zou, X., Gong, Y., Single-Atom Pt Anchored on Oxygen Vacancy of Monolayer Ti3C2Tx for Superior Hydrogen Evolution, **2022**, *Nano Lett.*, 22, 1398-1405, <https://doi.org/10.1021/acs.nanolett.1c04809>.
